# The galectin-3 inhibitor selvigaltin reduces liver inflammation and fibrosis in a high fat diet rabbit model of metabolic-associated steatohepatitis

**DOI:** 10.3389/fphar.2024.1430109

**Published:** 2024-07-31

**Authors:** Paolo Comeglio, Giulia Guarnieri, Sandra Filippi, Ilaria Cellai, Gabriele Acciai, Ian Holyer, Fredrik Zetterberg, Hakon Leffler, Barbro Kahl-Knutson, Erica Sarchielli, Annamaria Morelli, Mario Maggi, Robert J. Slack, Linda Vignozzi

**Affiliations:** ^1^ Department of Experimental and Clinical Biomedical Sciences “Mario Serio”, University of Florence, Florence, Italy; ^2^ Department of Experimental and Clinical Medicine, University of Florence, Florence, Italy; ^3^ Department of Neurosciences, Psychology, Drug Research and Child Health (NEUROFARBA), University of Florence, Florence, Italy; ^4^ Galecto Biotech AB, Copenhagen, Denmark; ^5^ Department of Laboratory Medicine, Lund University, Lund, Sweden; ^6^ Interuniversity Consortium “Istituto Nazionale Biostrutture e Biosistemi” (INBB), Rome, Italy

**Keywords:** metabolic syndrome, liver metabolism, fibrosis, inflammation, galectin, MASH, galectin-3 inhibitor, selvigaltin

## Abstract

**Introduction:**

Galectin-3 is a pro-fibrotic β-galactoside binding lectin highly expressed in fibrotic liver and implicated in hepatic fibrosis. Selvigaltin (previously known as GB1211) is a novel orally active galectin-3 small molecule inhibitor that has high affinity for galectin-3 (human K_D_ = 25 nM; rabbit K_D_ = 12 nM) and high oral bioavailability in rabbits and man. In this study the efficacy of selvigaltin was investigated in a high fat diet (HFD) rabbit model of metabolic-associated steatohepatitis (MASH).

**Methods:**

Male New Zealand White rabbits were individually caged under standard conditions in a temperature and humidity-controlled room on a 12 h light/darkness cycle. After 1 week of regular diet (RD), rabbits were randomly assigned for 8 or 12 weeks to different groups: RD/vehicle, RD/selvigaltin, HFD (8 weeks), HFD/vehicle and HFD/selvigaltin (0.3, 1.0, 5.0 or 30 mg/kg selvigaltin with vehicle/selvigaltin *p.o.* dosed therapeutically *q.d.* 5 days per week from week 9 or 12). Liver inflammation, steatosis, ballooning, and fibrosis was measured via blood metabolic markers, histomorphological evaluation [Oil Red O, Giemsa, Masson’s trichome, picrosirius red (PSR) and second harmonic generation (SHG)], and mRNA and protein expression.

**Results:**

Steatosis, inflammation, ballooning, and fibrosis were all increased from RD to HFD/vehicle groups. Selvigaltin demonstrated target engagement by significantly decreasing galectin-3 levels in the liver as measured via immunohistochemistry and mRNA analysis. Selvigaltin dose-dependently reduced biomarkers of liver function (AST, ALT, bilirubin), inflammation (cells foci), and fibrosis (PSR, SHG), as well as decreasing the mRNA and protein expression of several key inflammation and fibrosis biomarkers (e.g., IL6, TGFβ3, SNAI2, collagen). Doses of 1.0 or 5.0 mg/kg demonstrated consistent efficacy across most biological endpoints supporting the current clinical doses of selvigaltin being investigated in liver disease.

**Discussion:**

Selvigaltin significantly reduced hepatic inflammation and fibrosis in an HFD rabbit model of MASH following therapeutic dosing for 4 weeks in a dose-dependent manner. These data support the human selvigaltin dose of 100 mg *b.i.d.* that has been shown to reduce key liver biomarkers during a clinical study in liver cirrhosis.

## 1 Introduction

Galectins are proteins able to bind to carbohydrates and participate in several physiological processes, including cell migration, immune responses, and cell-to-cell interactions. Many studies show a significant increase in galectin-3, a ubiquitous beta-galactoside-binding protein involved in chronic inflammation and tissue fibrosis, in patients with fibrosis in various organs (Slack et al., 2021). In particular, galectin-3 has been associated with liver fibrosis (Hsu et al., 1999; Henderson et al., 2006; Gudowska et al., 2015; Mackinnon et al., 2023). Galectin-3 is the only chimeric protein with a C-terminal Carbohydrate Recognition Domain (CRD) linked to a proline, glycine, and tyrosine rich additional N-terminal domain. It is able to undergo oligomerization via both the N-terminal and CRD (Barondes et al., 1994; Lepur et al., 2012), resulting in the formation of galectin-3 molecules with multivalent CRDs, cross-linking oligosaccharides on the cell surface. This results in the formation of what has been described as a dynamic galectin lattice (Ahmad et al., 2004; Nabi et al., 2015), orchestrating its pleiotropic effects on a gamut of physiological processes (Troncoso et al., 2023).

In multiple models of organ fibrosis, galectin-3 has been proven to be potently pro-fibrotic (Slack et al., 2021). Although the macrophage is the driving cell type for galectin-3 production, other key fibrotic cell types, epithelial cells and myofibroblasts, can function as further sources of the lectin upon activation. The effects of galectin-3 on these cell types in relation to pro-fibrotic stimulation are broad and have been shown to include epithelial-mesenchymal-transition (EMT), resulting in increased production of extracellular matrix (Slack et al., 2021). Galectin-3, therefore, represents a new potential target, and its inhibition could be regarded as a promising antifibrotic therapeutic strategy.

The metabolic syndrome (MetS) is a constellation of metabolic abnormalities, including impaired glucose tolerance, insulin resistance, hypertension, and visceral obesity. In addition, MetS is also characterized by dyslipidemia (Després and Lemieux, 2006). Metabolic dysfunction-associated steatotic liver disease (MASLD), also known as non-alcoholic fatty liver disease (NAFLD), a pathophysiological accumulation of lipids within the liver, is considered the hepatic hallmark of insulin resistance associated to MetS and visceral obesity (Kitade et al., 2017; Stefan et al., 2019). The term MASLD covers a spectrum of histological findings ranging from simple steatosis to metabolic-associated steatohepatitis (MASH), formerly known as nonalcoholic steatohepatitis (NASH), the most severe form of MASLD, which can lead to cirrhosis and hepatocarcinoma (Boccatonda et al., 2023). Our group developed a non-genomic, high fat diet (HFD)-induced, rabbit model of MetS that closely recapitulates the human MetS phenotype (Filippi et al., 2009; Maneschi et al., 2013; Vignozzi et al., 2014; Comeglio et al., 2018; Sarchielli et al., 2020; Comeglio et al., 2021). We demonstrated that feeding rabbits a HFD for 12 weeks could induce MetS in most animals (70%), while the MetS condition is present in only 1% of the regular diet rabbits (Corona et al., 2021). The use of high fat diet in other species, including mice, induces only some components of MetS, but others such as hypertension, hyperglycemia, and/or insulin resistance are often absent, whereas New Zealand white rabbits consistently show compelling MetS hallmarks (Arias-Mutis et al., 2017; Arias-Mutis et al., 2018; Lozano et al., 2019).

Feeding rabbits a high fat diet (HFD; standard diet implemented with 0.5% cholesterol and 2.5% vegetable fat) for 12 weeks induced all the components of MetS, including hypertension, hyperglycemia, increased visceral fat mass, hyperlipidemia, as well as severe histological alterations within the liver associated with MASH, including mononuclear cell infiltrates, lipid accumulation and fibrosis (Maneschi et al., 2013; Vignozzi et al., 2014; Comeglio et al., 2018; Comeglio et al., 2021), . all hepatic hallmarks of insulin resistance in MetS (Bril et al., 2017; Kitade et al., 2017; Stefan et al., 2019). A common paradigm between human and animal models in the development of MASH is the hepatic accumulation of lipids secondary to high fat diet, obesity, and insulin resistance, with activation of inflammatory cascades and fibrogenesis (Buzzetti et al., 2016). HFD is also known to induce a significant increase of the mRNA expression of several pro-fibrotic markers in liver homogenates (Vignozzi et al., 2014; Comeglio et al., 2018). A visible collagen deposition forming pro-fibrotic septa has been reported at sites where fatty degeneration of hepatocytes occurred (Vignozzi et al., 2014).

HFD rabbits also showed insulin resistance, as proven by a higher area under the curve (AUC) for glycemia following an oral glucose challenge, as compared with rabbits fed a regular diet (RD) (Filippi et al., 2009; Maneschi et al., 2013; Vignozzi et al., 2014; Comeglio et al., 2018). The visceral adipose tissue (VAT) isolated from these rabbits is characterized by insulin-resistant preadipocytes with impaired lipid handling, mitochondrial function and adipogenesis (Maneschi et al., 2012) as well as prostatitis (Morelli et al., 2013) and several alterations of the skeletal muscle, as demonstrated by histochemical and molecular analysis of the quadriceps femoris muscle from RD and HFD rabbits (Sarchielli et al., 2020). Furthermore, HFD-induced metabolic derangements and hypothalamic metaflammation were associated with an impairment in the neurotransmitter network controlling GnRH, thus elucidating the pathogenic link between MetS and hypogonadotropic hypogonadism (Morelli et al., 2014; Sarchielli et al., 2021).

The aim of this study was to assess tolerability and pharmacokinetics (PK) of the clinical galectin-3 inhibitor selvigaltin [known previously as GB1211, an alpha-monogalactoside with high affinity and selectivity for galectin-3 (Zetterberg et al., 2022; Aslanis et al., 2023), in an acute dosing regimen in the final week of the HFD rabbit model (week 12). This enabling study would also allow the determination of galectin-3 and related biomarkers using different tools, to observe if values are elevated in HFD rabbits compared with RD rabbits and validate the suitability of the model for further investigation of chronic treatment studies with selvigaltin. Subsequently, a study was carried out using a selvigaltin therapeutic dosing regimen over the final 4 weeks of HFD to evaluate the efficacy of the galectin-3 inhibitor in MASH and fibrosis across multiple endpoints, including effects on an extensive set of inflammatory and fibrotic markers, with analyses of liver inflammation, steatosis, ballooning, and fibrosis by blood metabolic markers, histomorphological evaluation [Oil Red O, Giemsa, Masson’s trichome, picrosirius red (PSR) and second harmonic generation (SHG)], as well as mRNA and protein expression of several key inflammation and fibrosis biomarkers.

## 2 Materials and methods

### 2.1 Experimental plan

A total number of 64 male New Zealand White rabbits (Charles River, Calco, Lecco, Italy), weighing about 3 kg, were individually caged under standard conditions in a temperature- and humidity-controlled room on a 12 h light/dark cycle. Water and food were unrestricted throughout the study. Experimental procedures were conducted using the facilities of the Department of Biomedical Experimental and Clinical Sciences “Mario Serio” and Department of Experimental and Clinical Medicine, and those of CE.S.A.L. (Centro Stabulazione degli Animali da Laboratorio), and NEUROFARBA Department, University of Florence, Italy.

As a preliminary trial - to test the galectin-3 inhibitor selvigaltin in a full 12-week HFD study - an acute 1-week *in vivo* treatment protocol was designed to evaluate the tolerability and PK of selvigaltin in its optimum vehicle formulation (PEG300/Solutol, 90:10). The dose employed was established based on pilot pharmacokinetics studies in rabbits performed historically in naïve rabbits (data not shown). This first part of the study allowed the determination of the therapeutic benefits of selvigaltin following acute treatment, thus validating the model for further investigation of this mechanism in a chronic treatment protocol.

After 1 week of standard diet, animals (n = 12) were randomly assigned to the following groups (Table 1A):

• Control rabbits continued to receive a regular diet (RD) for 12 weeks and were treated for 5 days (days 1–5, p.o.), during the last week before culling, with either vehicle; RD+1W Veh group] or GB1211 (30 mg/kg; RD + 1W 30 mg group);
• Rabbits received a high fat diet (HFD) for 12 weeks (RD implemented with 0.5% cholesterol and 2.5% vegetable fat) and were treated for 5 days (days 1–5, p.o.), during the last week before culling, with either vehicle (HFD + 1W Veh group) or GB1211 (30 mg/kg; HFD + 1W 30 mg group).

**TABLE 1:** Experimental design of HFD rabbit studies with selvigaltin. (Panel A) - HFD rabbit study–acute 5-day selvigaltin treatment at week 12. (Panel B) - HFD rabbit study–prolonged, multiple dose selvigaltin treatment at weeks 9–12.

Group	Diet	Dose *q.d.* Days 1–5	Dose	Dosing schedule	Group size (n)
RD+1W Veh	RD	Vehicle (*p.o.*)	-	Week 12	3
RD+1W 30 mg	RD	Selvigaltin (*p.o.*)	30 mg/kg	Week 12	3
HFD+1W Veh	HFD	Vehicle (*p.o.*)	-	Week 12	3
HFD+1W 30 mg	HFD	Selvigaltin (*p.o.*)	30 mg/kg	Week 12	3

Group	Diet	Dose *q.d*. Days 1–5	Dose	Dosing schedule	Group size (n)
RD+4W Veh	RD	Vehicle (*p.o.*)	-	Weeks 9–12	6
HFD 8W	HFD	-	-	Culled at week 8 prior to dosing (baseline fibrosis)	6
HFD+4W Veh	HFD	Vehicle (*p.o.*)	-	Weeks 9–12	8
HFD+4W 0.3 mg	HFD	Selvigaltin (*p.o.*)	0.3 mg/kg	Weeks 9–12	8
HFD+4W 1.0 mg	HFD	Selvigaltin (*p.o.*)	1.0 mg/kg	Weeks 9–12	8
HFD+1W 30 mg	HFD	Selvigaltin (*p.o.*)	30 mg/kg	Weeks 9–12	8

RD, regular diet; HFD, high fat diet; W, week; Veh, vehicle; *q.d.*, once daily; *p.o.*, oral gavage.

The second part of the study evaluated the effects on HFD rabbits of a prolonged 4-week *in vivo* treatment with selvigaltin, to investigate the therapeutic benefits of the compound over a chronic dosing regimen. After 1 week (W) of RD, the animals (n = 44) were randomly assigned to the following groups (Table 1B):

• Control rabbits continued to receive a RD for 12 weeks and were treated with vehicle for 5 days a week (days 1–5, p.o.) during the last 4 weeks before culling (RD + 4W Veh group);
• Rabbits received a HFD for 8 weeks and were culled (HFD 8W group);
• Rabbits received a HFD for 12 weeks and were treated with vehicle for 5 days a week (days 1–5, p.o.) during the last 4 weeks before culling (HFD+4W Veh group);
• Rabbits received a HFD for 12 weeks and were treated with GB1211 (0.3 mg/kg) for 5 days a week (days 1–5, p.o.) during the last 4 weeks before culling (HFD + 4W 0.3 mg group);
• Rabbits received a HFD for 12 weeks and were treated with GB1211 (1.0 mg/kg) for 5 days a week (days 1–5, p.o.) during the last 4 weeks before culling (HFD + 4W 1.0 mg group);
• Rabbits received a HFD for 12 weeks and were treated with GB1211 (5.0 mg/kg) for 5 days a week (days 1–5, p.o.) during the last 4 weeks before culling (HFD + 4W 5.0 mg group).

Before reaching the end of treatment one animal from each of the 4-week HFD vehicle and selvigaltin dosing groups died prematurely. As the same animal loss was observed across groups, we believe it is likely a result of HFD diet and excessive lipid content in the blood with no trend implicating a role of study drug. Rabbits were sacrificed by a lethal dose of sodium thiopental [200 mg/kg intravenous (i.v.)], and liver, heart, kidneys, visceral adipose tissue, prostate, and seminal vesicles were harvested, weighed, and stored at −80°C for subsequent analyses.

### 2.2 Ethical statement

All animals received human care and the animal handling complied with the Animal Welfare Body of the University of Florence, Florence, Italy, in accordance with the Italian Ministerial Law n. 26/2014. The study was approved by the Ministry of Health authorization n. 146/2021-PR.

Animal experiments conformed to the Animal Research: Reporting of *In Vivo* Experiments (ARRIVE) guidelines (http://www.nc3rs.org.uk/arrive-guidelines).

### 2.3 Biochemical and metabolic analyses

The oral glucose tolerance test (OGTT) was performed in accordance with the published method (Filippi et al., 2009). Briefly, after an overnight fast, a 50% glucose solution was orally administered to the animals at a dose of 1.5 g/kg. Blood samples were collected before and 15, 30, and 120 min after glucose loading. The incremental area under the curve (iAUC) was calculated by using GraphPad Prism (GraphPad Software, San Diego, CA). Blood samples for analyses were obtained from the marginal ear vein at week 8 or 12, in all groups. All blood samples were collected in standard conditions, before 10 a.m. after an overnight fasting. The blood was immediately centrifuged at 2,000 *g* for 20 min at 4°C and collected plasma/serum stored at −80°C until assayed. Plasma glucose, total cholesterol, triglycerides, transaminases (AST and ALT), and gamma-glutamyl transferase levels were measured using an automated system (Cobas 8000, Roche Diagnostics International AG, Rotkreuz, Switzerland). Serum alkaline phosphatase, albumin and total bilirubin levels were measured at IDEXX Laboratories (Kornwestheim, Germany), using an automated system (AU480, Beckman-Coulter, Brea, CA) with kinetic and photometric colour tests *as per* the following methods: alkaline phosphatase - alkaline buffer solution [2-amino-2-methylpropanol (AMP) in conjunction with p-nitrophenyl phosphate (pNPP)], according to the recommendation of the International Federation for Clinical Chemistry; albumin - the reaction of bromocresol green with albumin produces a colour complex, the absorption of which is measured dichromatically (Doumas et al., 1971); total bilirubin - a stabilized diazonium salt, 3,5-dichlorophenyldiazonium tetrafluoroborate (DPD), reacts with bilirubin to formazobilirubin, which absorbs at 570 nm (Tolman and Rej, 1999).

Mean arterial blood pressure (MAP) was measured by using a polyethylene catheter inserted into a femoral artery at week 8 or 12, after ketamine (10 mg/kg i.v.) and sodium thiopental (50 mg/kg i.v.) sedation.

### 2.4 Galectins fluorescence polarization binding assay

Rabbit recombinant galectin was produced as previously described (Sörme et al., 2004). Galectin fluorescence polarization (FP) binding assays were completed as previously described (Sörme et al., 2004). Briefly, FP was measured from above in 96-well microtiter plates (black polystyrene; Costar, Corning, NY) using a POLARStar^®^ instrument (BMG Labtech, Ortenberg, Germany). The final sample volume in each well was 200 μL. For inhibition assays, 100 μL of galectin and probe at fixed concentration was mixed with 100 μL of inhibitor solution and FP was measured as described above. Control wells containing only fluorescent probe or fluorescein were included.

All dilutions and measurements were done in PBS with plates incubated at room temperature for 1 h. K_D_ values for galectin–inhibitor interactions were calculated directly from single data points by solving the two equations of mass action governing galectin–inhibitor interaction and galectin–probe interaction as previously described (Sörme et al., 2004).

### 2.5 Plasma and liver selvigaltin bioanalysis

The *in vitro* (tested at nominal concentrations of 0.2, 2, and 20 μg/mL) and *ex vivo* plasma protein binding of selvigaltin in rabbit blood was measured by rapid equilibrium dialysis and LC-MS/MS.

Plasma and liver homogenate concentrations of selvigaltin were determined at Red Glead Discovery AB (Lund, Sweden) by LC-MS/MS using a QTRAP6500 (plasma) or QTRAP4500 (liver) mass spectrometer (AB Sciex LLC, Framingham, MA). Liver homogenates were prepared by homogenisation of 200 mg liver tissue/ml water using a Fisherbrand™ Homogenizer 150 (Thermo Fisher Scientific, Waltham, MA). Plasma or liver homogenate were mixed 1 part matrix with 3 parts acetonitrile prior to centrifugation at 10,000 *g* for 10 min at 18°C with supernatant diluted x4 with water prior to LC-MS/MS analysis.

### 2.6 Liver histomorphology

Liver specimens were fixed in 10% buffered formalin (Sigma-Aldrich Corp., St. Louis, MO), paraffin embedded and sectioned at a thickness of 5 μm with a microtome. The slides were stained with hematoxylin and eosin (H&E; both Bio-Optica, Milan, Italy), following standard protocols for histological analysis, to evaluate the general morphology and tissue structure, and then analyzed to evaluate inflammation and steatosis/ballooning using Giemsa and Masson’s trichrome staining, respectively. Briefly, deparaffinized and rehydrated sections were incubated with Giemsa (Bio-Optica) in distilled water at ratio 1:1 or with Masson’s trichrome (Bio-Optica), following the manufacturer’s instructions, as previously described (Vignozzi et al., 2014). To evaluate lipid accumulation, liver specimens were also cryosectioned at a thickness of 8 μm, fixed with 4% paraformaldehyde, treated for 2–5 min with isopropanol and stained with Oil Red O (Bio-Optica) for 20 min, following the manufacturer’s instructions.

In liver, collagen content evaluation for fibrosis grade and quantification was conducted by staining using Picrosirius Red Stain kit (Bio-Optica), as per manufacturer’s instructions. Briefly, deparaffinized and rehydrated sections were incubated with the staining solution for 50 min, rinsed with the appropriate reagents and with distilled water, dehydrated through ascending alcohols and xylene, and mounted, as previously described (Comeglio et al., 2017).

The expression of RAM11, galectin-3, and vimentin was evaluated by immunohistochemical analysis on 5 μm thick paraffin embedded liver sections. Briefly, deparaffinized and rehydrated sections were incubated overnight at 4°C with mouse monoclonal anti-RAM11 (1:80; Dako, Carpenteria, CA), mouse monoclonal anti-galectin-3 (1:100; Abcam, Cambridge, United Kingdom) or mouse monoclonal anti-vimentin (1:800; Santa Cruz Biotechnology, Dallas, TX) antibodies, followed by biotinylated anti-mouse secondary antibody (Millipore, Burlington, MA) and streptavidin-peroxidase complex (Millipore). The reaction product was developed with 3′,3′-diaminobenzidine tetrahydrochloride as chromogen (Merck KgaA, Darmstadt, Germany). Negative controls were performed avoiding primary antibody. Percentage of the sampled area for galectin-3 and vimentin staining was obtained on ×200 and ×40 original magnification slides, respectively, using ImageJ software on five fields for each slide. Semi-quantitative staining of percentage of the sampled area for Picrosirius Red, Oil Red O, galectin-3 and vimentin was obtained using open source Java-based ImageJ software (Fiji bundle, https://imagej.net/). All slides were evaluated blindly and photographed using a Nikon Microphot-FXA microscope (Nikon, Tokyo, Japan).

#### 2.6.1 TPE/SHG imaging of liver tissues

Blinded images of unstained sections of liver were acquired using a Genesis^®^200 imaging system (HistoIndex, Singapore) and analyzed by Second Harmonic Generation (SHG) microscopy to visualize collagen and Two-Photon Excitation Fluorescence (TPEF) microscopy to visualize the other tissue structures as previously described (Liu et al., 2017).

Briefly, deparaffinized tissue sections were imaged by SHG at 780 nm (specific to collagen-1 and -3) simultaneously with TPEF at 780 nm (collected at 550 nm to visualize tissue structure). Both were captured at a ×20 objective and 0.4 µM resolution. A specialized artificial intelligence analysis program developed by, and proprietary to, HistoIndex were employed to identify and quantify collagen fibres across liver regions with outputs highlighted below and as previously described (Liu et al., 2017):

• SHG Overall Area (Percentage of total collagen in overall region).
• Strings Overall N. (Number of collagen strings in overall region).
• Strings Overall Length (Total length of all collagen strings in overall region).
• Strings PT Area (Total area of all collagen strings in portal tract region).
• Strings PT Length (Total length of all collagen strings in portal tract region).
• Strings PS Area (Total area of all collagen strings in peri-sinusoidal region).
• Strings PS Length (Total length of all collagen strings in peri-sinusoidal region).

#### 2.6.2 Immunofluorescence

Co-expression of galectin-3 with the stellate cell marker vimentin or the macrophagic marker RAM11 was evaluated in vehicle-treated HFD rabbit liver biopsies by immunofluorescence analysis. Briefly, paraffin-embedded sections (5 μm) were deparaffinized, rehydrated, boiled for 10 min in 10 mM, pH 6.0 sodium citrate buffer (Zitomed System, Berlin, Germany) for antigen retrieval, and then incubated in blocking solution (PBS, albumin bovine serum 1%), at room temperature for 45 min. The sections were subsequently incubated with goat polyclonal anti-galectin-3 (1:100; Abcam) and mouse monoclonal anti-vimentin (1:1000; Santa Cruz Biotechnology) or mouse monoclonal anti-RAM11 (1:100; Dako) primary antibodies, overnight at 4°C. The following day, slides were incubated in the dark with donkey anti-goat and anti-mouse (1:200; both Thermo Fisher Scientific) IgG secondary antibodies, labelled with Alexa Fluor 488 and 568, respectively. Nuclei were counterstained using ProLong Gold antifade reagent with DAPI (Thermo Fisher Scientific). Negative controls were performed avoiding the primary antibodies.

Representative images were acquired with a Nikon Microphot-FXA microscope (Nikon). The number of double-positive (galectin-3/RAM11) cells was counted in 6 fields for each slide and expressed as percentage of RAM11 positive cells.

### 2.7 RNA extraction and RT-PCR analysis

TRIzol reagent (Life Technologies, Paisley, United Kingdom) and/or RNeasy Mini Kit (Qiagen, Hilden, Germany) were used to isolate total RNA from rabbit liver specimens. RNA concentration and quality were measured using a Nanodrop ND-1000 (Thermo Fisher Scientific) at 260 nm and 280 nm. The OD 260/280 absorbance ratios were between 2.0 and 2.1 for all samples, to confirm RNA integrity and purification. cDNA synthesis was carried out using the iScript™ cDNA Synthesis Kit (Bio-Rad Laboratories, Hercules, CA), with 100 ng of mRNA in 20 µL reaction volume in accordance with the following protocol: 5 min at 25°C, 20 min at 46°C and 5 min at 85°C, followed by refrigeration at 4°C.

Semi-quantitative real-time RT-PCR (qRT-PCR) amplification and detection was performed with SsoAdvanced™ universal SYBR^®^ Supermix and a CFX96 Two-Color Real-Time PCR Detection System (both Bio-Rad Laboratories) with the following thermal cycler conditions: 40 cycles at 95°C for 15 s and 60°C for 1 min. Specific PCR primers for rabbit target genes were designed on sequences available at the National Center for Biotechnology Information GenBank (http://www.ncbi.nlm.nih.gov) or Ensemble Genome (http://www.ensembl.org).

The 18S ribosomal RNA subunit was quantified with a predeveloped assay (Hs99999901_s1, Life Technologies) and used as the housekeeping gene for the relative quantitation of the target genes based on the comparative threshold cycle (Ct) 2^−ΔΔCt^ method (Livak and Schmittgen, 2001), with some modifications. In detail, we used the vehicle control groups as the calibrator in each analysis, so that the calculations would provide the fold-change of the other groups relative to controls.

### 2.8 Protein expression in liver homogenates

Quantitative analyses of collagen, IL6 and TNFα expression in liver biopsies were carried out using available commercial assays. Collagen content was evaluated in liver homogenates employing the Sircol S2000 assay (Biocolor, Carrickfergus, United Kingdom), in accordance with manufacturer’s protocols. Briefly, 30 mg liver biopsies were fragmented in the appropriate reagent and the supernatant was bound to a dye reagent containing Sirius Red, which would generate a colorimetric reaction quantified as absorbance using an internal collagen standard curve. IL6 and TNFα were quantified in liver homogenates using ELISA assay kits (both from Cloud-Clone Corp, Katy, TX). Briefly, 50 mg liver biopsies were homogenized in proprietary lysis buffer and the supernatants were analyzed by sandwich immunoassays, following the producer’s instructions.

### 2.9 Statistical analysis

All data were obtained from at least three independent experiments. Statistical analysis was performed using Shapiro-Wilk test of normality, and then one-way non-parametric ANOVA Kruskal–Wallis test followed by *post hoc* Dunn’s analysis (for not normally distributed parameters) or one-way parametric ANOVA test followed by *post hoc* Fisher’s Least Significant Difference (LSD) test analysis (for normally distributed parameters), to evaluate differences between groups, with *p* < 0.05 considered as significant. Contingency tables analyses were used for binomial or polynomial variables, with Pearson’s chi-squared test used for statistical comparisons and *p* < 0.05 considered as significant. Correlations were evaluated by Spearman bivariate correlation analyses. Statistical analysis was performed with Statistical Package for the Social Sciences (v.29.0.2; SPSS Inc., Chicago, IL) or GraphPad Prism (v.8.4; GraphPad Software).

## 3 Results

### 3.1 HFD rabbit study – acute 5-day selvigaltin treatment at week 12

#### 3.1.1 Selvigaltin pharmacokinetics

Preliminary experiments showed that selvigaltin bound to rabbit galectin-3 with a K_D_ value of 12 ± 3 nM (mean ± SD; n = 3), equivalent to a concentration of 6.4 ng/mL. This finding is comparable to the reported human galectin-3 affinity of 25 nM (Zetterberg et al., 2022). Conversely, the binding affinity of selvigaltin for mouse galectin-3 (770 nM) is ∼31-fold lower compared with human and therefore makes translational comparisons of PK/PD from mouse to human not possible (Zetterberg et al., 2022).

From *in vitro* plasma protein binding in rabbit blood, the unbound free fraction of selvigaltin was determined to be 14.1%. From *ex-vivo* samples, taken at terminal bleed after 5 days of acute selvigaltin dosing in RD and HFD rabbits at week 12 of the model, plasma protein binding was determined to be 5.4% and 7.1%, respectively.

**FIGURE 1:**
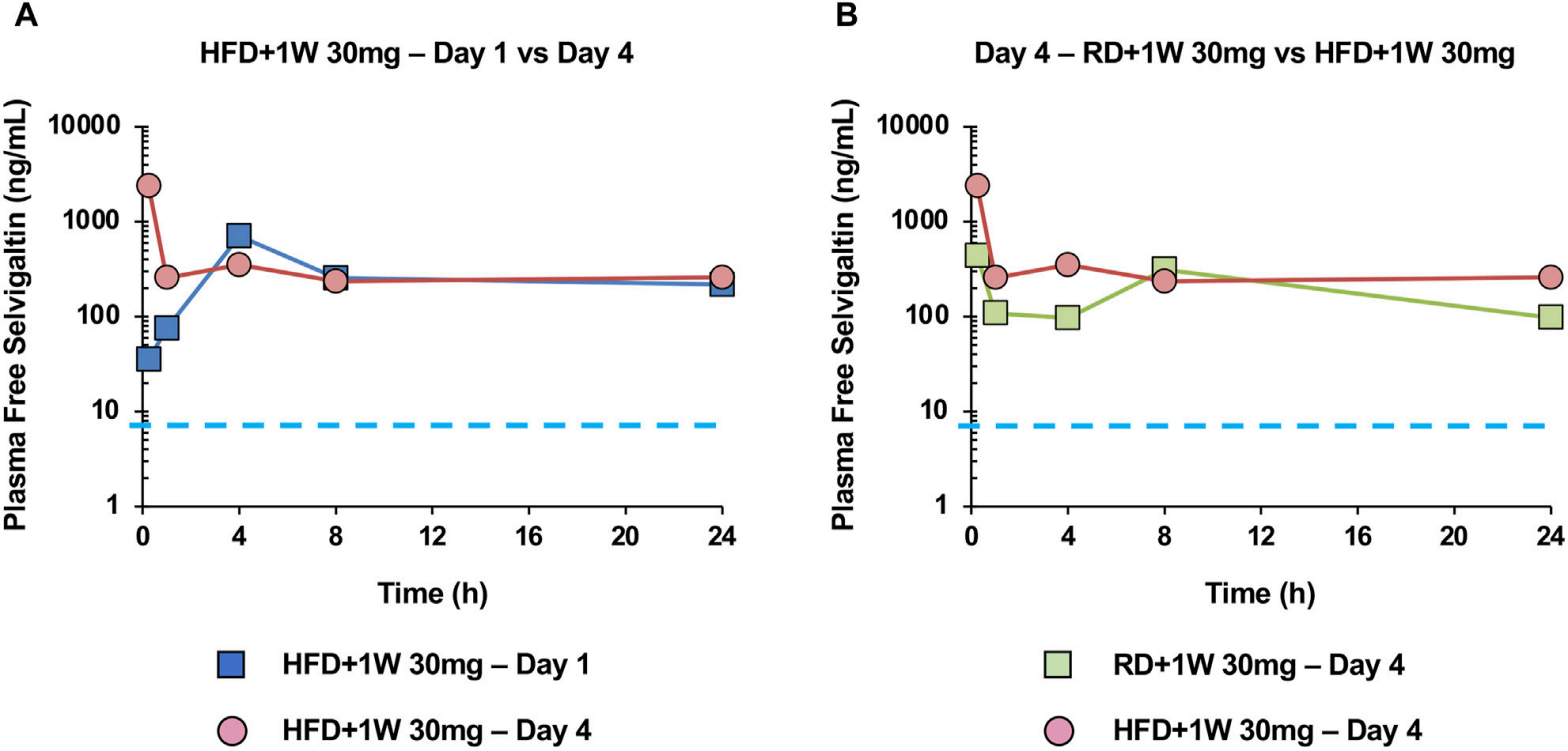
Selvigaltin pharmacokinetics in rabbit plasma during treatment. Panel **(A)** shows plasma free selvigaltin over the 24 h at Day 1 and Day 4 in HFD rabbits treated with the compound (n = 3). Panel **(B)** shows plasma free selvigaltin over the 24 h at Day 4 in RD (n = 3) and HFD (n = 3) rabbits treated with the compound. Dashed line indicates the rabbit galectin-3 K_D_ (6.4 ng/mL).

Figure 1A shows the time course (hours) of plasma free selvigaltin in HFD+1W 30 mg rabbits after 1 or 4 days of treatment. At time 0 of day 1, plasma concentrations of selvigaltin showed a high free systemic exposure of drug that was over the rabbit galectin-3 K_D_ at steady state (i.e. 6.4 ng/mL, dashed line). The free systemic exposure rose consistently in the subsequent hours and was maintained during the course of the treatment from Day 1 to Day 4 (Figure 1A).

Figure 1B shows the comparison between exposure of selvigaltin in treated rabbit fed or not with HFD, at day 4 of treatment. A marginal increase in exposure was observed for selvigaltin in HFD+1W 30 mg animals, compared to the RD+1W 30 mg group (Figure 1B), that was used to guide the dose selection in the prolonged *in vivo* treatment part of the study. No difference between selvigaltin plasma protein binding was observed between RD and HFD rabbits, suggesting that HFD diet had no effect on this parameter.

#### 3.1.2 Effects of acute selvigaltin treatment on metabolic and biochemical parameters

Table 2 shows biochemical and metabolic data from the four experimental groups: RD + 1W Veh, RD + 1W 30 mg, HFD + 1W Veh, and HFD + 1W 30 mg rabbits. HFD induced hyperglycaemia, impaired glucose tolerance, hyperlipidaemia, hypertension, increased liver weight, and elevated transaminases, as expected (Comeglio et al., 2018). Selvigaltin-treated HFD rabbits, when compared to vehicle-treated ones, showed a significant reduction in plasma cholesterol (*p* < 0.001 vs. HFD + 1W Veh) and normalization of plasma triglycerides levels (*p* < 0.05 vs. HFD + 1W Veh). In addition, selvigaltin reduced the HFD-induced increase in ALT plasma levels (Table 2; *p* = 0.059 vs. HFD+1W Veh).

**TABLE 2:** Clinical and biochemical data at sacrifice.

Variable	RD+1W Veh	RD+1W 30 mg	HFD+1W Veh	Sign.	HFD+1W 30mg	Sign.
*Glycaemia (gr/L)*	*1.33 ± 0.13*	*1.12 ± 0.17*	*1.42 ± 0.19*		*1.60 ± 0.14*	***°°***
**OGTT (iAUC)**	**156.83 ± 4.47**	**165.90 ± 11.12**	**202.03 ± 12.41**	*** °°	**220.33 ± 8.63**	*** °°° ^
**Plasma Cholesterol (mg/dL)**	**27.33 ± 6.43**	**21.67 ± 3.51**	**3368.00 ± 423.26**	*** °°°	**2064.00 ± 268.00**	*** °°° ^^^
**Plasma Triglycerides (mg/dL)**	**82.00 ± 31.43**	**56.00 ± 21.28**	**260.67 ± 135.36**	* °°	**88.00 ± 42.14**	**^**
**MAP (mmHg)**	**88.33 ± 10.41**	**88.75 ± 14.09**	**120.83 ± 4.79**	* °	**130.83 ± 20.36**	** °°
**Liver Weight (% of BW)**	**2.45 ± 0.28**	**2.40 ± 0.33**	**3.78 ± 0.10**	** °°	**3.94 ± 0.55**	*** °°°
**AST (U/L)**	**33.67 ± 2.08**	**29.33 ± 5.86**	**165.67 ± 90.39**	** °°	**231.67 ± 22.30**	*** °°°
**ALT (U/L)**	**35.33 ± 6.43**	**36.33 ± 25.11**	**159.00 ± 32.05**	** °°	**97.33 ± 54.99**	

Results are reported as mean ± SD (standard deviation); n = 3 in all groups. iAUC, incremental area under the curve of glucose blood level during oral glucose tolerance test (OGTT); MAP, mean arterial pressure; BW, body weight; AST, aspartate aminotransferase; ALT, alanine aminotransferase. Significance (Sign.): one-way parametric ANOVA, test followed by *post hoc* Fisher’s Least Significant Difference (LSD) test for normally distributed data (in bold) and one-way non-parametric ANOVA, Kruskal–Wallis test followed by *post hoc* Dunn’s analysis for not normally distributed data (in italic). *p < 0.05, **p < 0.01, ***p < 0.001 vs. RD+1W Veh; ° *p* < 0.05, °° *p* < 0.01, °°° *p* < 0.001 vs. RD+1W 30mg; ^ *p* < 0.05, ^^^ *p* < 0.001 vs. HFD+1W Veh.

#### 3.1.3 Immunohistochemistry assessment of liver steatosis, inflammation, ballooning, and fibrosis

**FIGURE 2:**
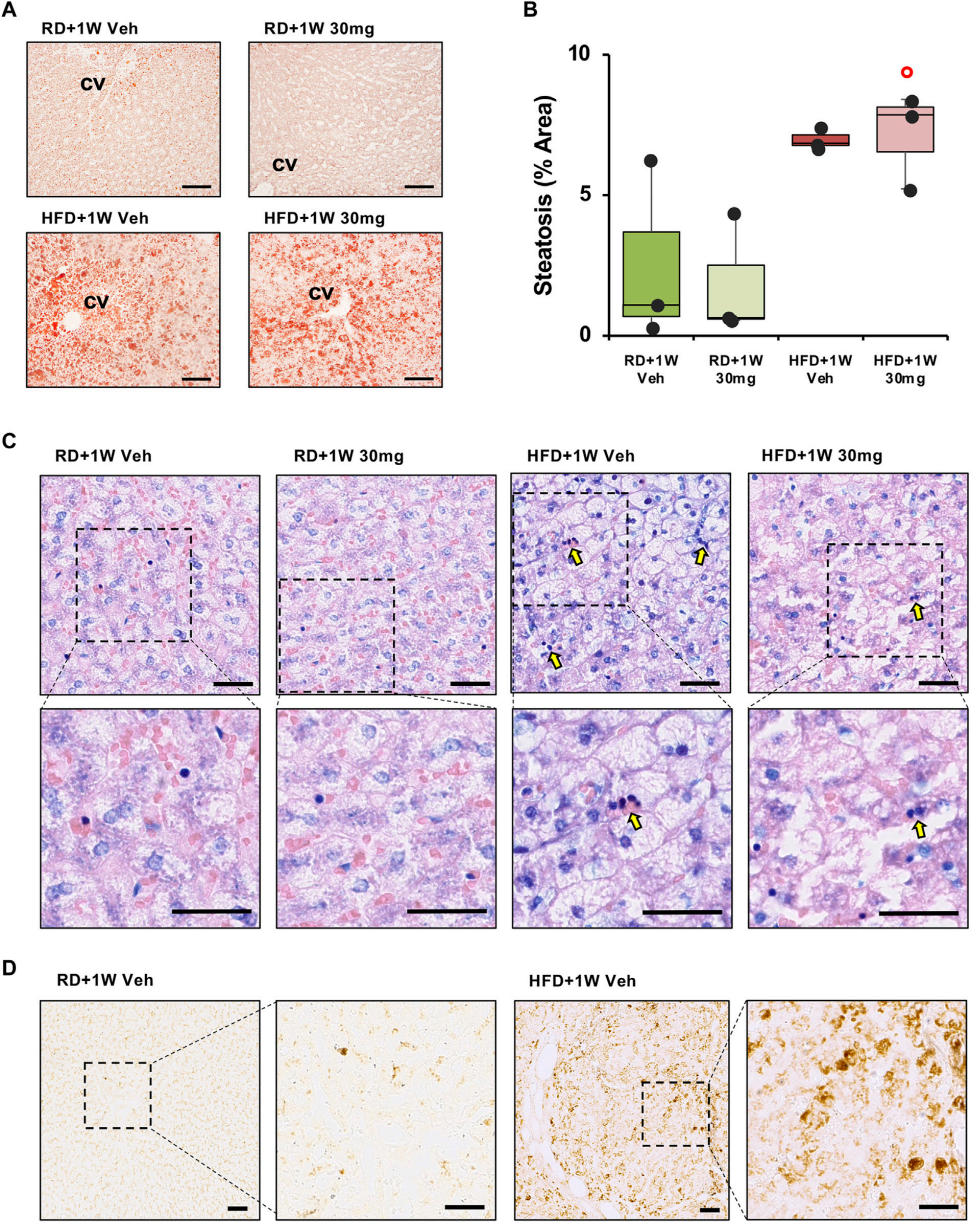
Oil Red O, Giemsa and RAM11 staining of liver sections. Panel **(A)** shows representative images of Oil Red O staining ×100 original magnifications (scale bar = 100 µm). Panel **(B)** shows quantification of the percentage of steatosis area. Significance was calculated using one-way non-parametric ANOVA Kruskal–Wallis test followed by *post hoc* Dunn’s analysis for not normally distributed data. ° *p* < 0.05 vs. RD + 1W 30 mg. Panel **(C)** shows inflammatory infiltration of liver specimens by Giemsa staining. Representative images of ×200 and ×400 original magnifications (n = 3 in all groups; scale bar = 50 µm). Arrows indicate foci of inflammatory infiltrates. Panel **(D)** shows macrophagic marker RAM11 immunodetection in liver sections in ×100 and ×200 original magnifications of representative images of RD+1W Veh and HFD+1W Veh samples (scale bar: 50 µm).

The extent of liver steatosis was established by staining with Oil Red O, a lysochrome fat-soluble diazo dye used for staining neutral triglycerides and lipids in frozen sections. We observed a marked increase in steatosis in both HFD groups compared to RD livers (Figures 2A,B).

We then evaluated treatment outcome in terms of anti-inflammatory effects of selvigaltin. Figure 2C shows representative images of ×200 original magnifications of Giemsa staining in the different experimental groups. Foci of inflammatory infiltrates (dark blue/purple nuclei) are mostly visible in the HFD + 1W Veh group (Figure 2C, arrows), but not in the HFD + 1W 30 mg group. Higher magnifications of Giemsa staining (Figure 2C) clearly show inflammatory infiltration in HFD+1W Veh samples. Validated rabbit macrophage marker RAM11 (Lis et al., 2010) immunostaining of placebo-treated RD and HFD liver biopsies further confirmed that inflammation in HFD specimens was mainly due to macrophage infiltration, as shown in Figure 2D (×100 and ×200 original magnifications).

**FIGURE 3:**
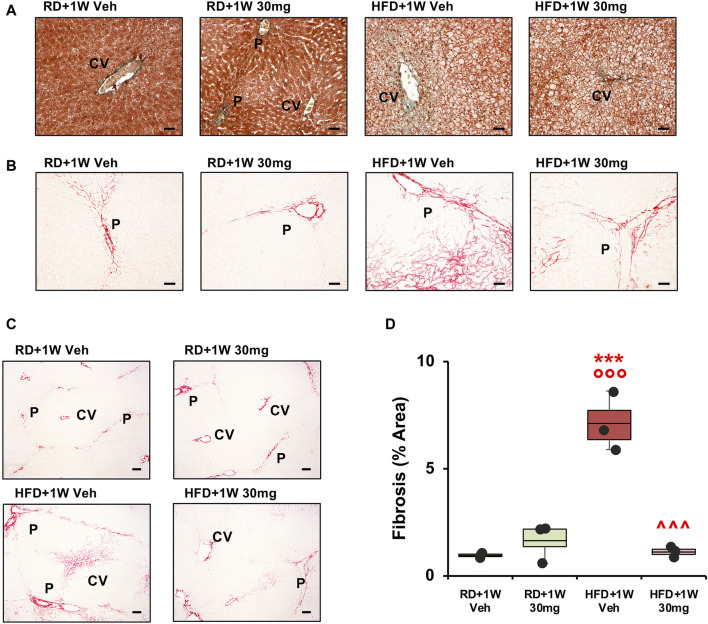
Masson’s trichrome and Picrosirius Red analyses of liver sections. Panel **(A)** shows representative images of Masson’s trichrome staining ×100 original magnifications (scale bar = 50 µm). Panel **(B)** shows PSR analysis of collagen deposition in rabbit liver sections evidencing the periportal space. The representative images at ×100 magnification (scale bar = 50 µm) show the fibrosis extension. Panel **(C)** shows representative images of biopsies at ×40 magnification (scale bar = 100 µm). P, portal vein; CV, centrilobular vein. Panel **(D)** shows percentage of fibrosis area in rabbit liver sections. Data are reported as box plots with data points (n = 3 in all groups). Significance was calculated using one-way parametric ANOVA test followed by *post hoc* Fisher’s Least Significant Difference (LSD) test for normally distributed data. ***p < 0.001 vs. RD+1W Veh; °°° *p* < 0.001 vs. RD+1W 30mg; ^^^ *p* < 0.001 vs. HFG + 1W Veh.

The presence of hepatocyte ballooning was observed by analyzing liver specimens with ×100 original magnifications of Masson’s trichrome staining. Figure 3A reports representative images from all rabbits, showing disarranged lobular structure and most hepatocytes presenting ballooning in HFD liver specimens, compared to a normal structure and lipid absence in RD samples, without any significant effect of selvigaltin.

Finally, we studied the HFD-induced effect on fibrosis by analyzing the samples with Picrosirius Red (PSR) staining. As established by observation of ×40 magnification slides, only a mild physiological structural deposition is observed in both RD groups, as already reported in rabbits (Lu et al., 2014). Figure 3B displays ×100 magnifications representative images of all groups. One-week treatment of HFD animals with selvigaltin markedly reduced collagen deposition, compared to vehicle-treated HFD rabbits. In particular, marked portal to portal bridges are visible in the HFD+1W Veh samples, where pericellular and sinusoidal fibrosis and the presence of a “chicken wire” pattern (typical of advanced stages of fibrosis) are also observable. These peculiar features are clearly reduced in the HFD+1W 30 mg group.

Figure 3C shows representative images at ×40 magnification of liver biopsies stained with PSR from all experimental groups, where portal to portal bridges and portal to central bridges are evident only in HFD + 1W Veh rabbits. Figure 3D bar graph reports the densitometry of the collagen deposition of the sampled areas in all rabbits. The mean percentage of fibrosis showed a significant 6-7-fold increase in HFD+1W Veh slides, compared to RD+1W Veh and RD+1W 30 mg groups (*p* < 0.001 and *p* < 0.01, respectively), and selvigaltin treatment in HFD rabbits significantly reduced fibrosis to RD levels (*p* < 0.001 vs. HFD + 1W Veh).

The grading system for inflammation enumerated the number of foci in each 200x field assessed, according to Kleiner et al. (2005): 0 = no foci; 1 = <2 foci; 2 = 2–4 foci; 3 => 4 foci. As shown in the contingency table (Table 3), statistically higher scoring grades were recorded in the HFD+1W Veh group, compared to RD+1W Veh and HFD + 1W 30 mg (both *p* < 0.05).

**TABLE 3:** Contingency tables for inflammation, ballooning, and fibrosis scoring.

Analyses	Contingency table scores	RD+1W Veh	RD+1W 30 mg	HFD+1W Veh	Sign	HFD+1W 30 mg	Sign
**Inflammation**	**0**	100.0%	66.7%	0.0%	*	100.0%	**^**
	**1**	0.0%	33.3%	66.7%		0.0%	
	**2**	0.0%	0.0%	33.3%		0.0%	
	**3**	0.0%	0.0%	0.0%		0.0%	
**Ballooning**	**0**	100.0%	100.0%	0.0%	* °	0.0%	* °
	**1**	0.0%	0.0%	33.3%		66.7%	
	**2**	0.0%	0.0%	66.7%		33.3%	
**Fibrosis (Ishak Score)**	**0**	0.0%	0.0%	0.0%	**# §**	0.0%	**ç**
	**1**	0.0%	0.0%	0.0%		0.0%	
	**2**	66.7%	33.3%	0.0%		33.3%	
	**3**	33.3%	66.7%	0.0%		66.7%	
	**4**	0.0%	0.0%	66.7%		0.0%	
	**5**	0.0%	0.0%	33.3%		0.0%	

Significance (Sign.): Pearson’s chi-squared test (n = 3 in all groups). *p < 0.05, #*p* = 0.112 vs. RD+1W Veh; ° *p* < 0.05, §*p* = 0.112 vs. RD+1W 30mg; ^ *p* < 0.05, ç *p* = 0.112 vs. HFD+1W Veh.

Ballooning analysis was based on the grading system reported by Kleiner et al. (2005): 0 = no ballooning; 1 = few ballooned cells; 2 = prominent ballooning. Data, reported in Table 3 as contingency table, shows a ballooning score decrease in HFD + 1W 30 mg animals, compared to the HFD + 1W Veh group, although the change is not statistically significant.

The results show consistent fibrosis in the HFD + 1W Veh samples analyzed, with the Ishak score evaluated in accordance with the literature (Ishak et al., 1995; Standish et al., 2006; Ghany et al., 2009) and reported in Table 3. HFD rabbits treated with selvigaltin display a marked decrease in fibrosis, with normalization of the Ishak score up to the RD levels.

**FIGURE 4:**
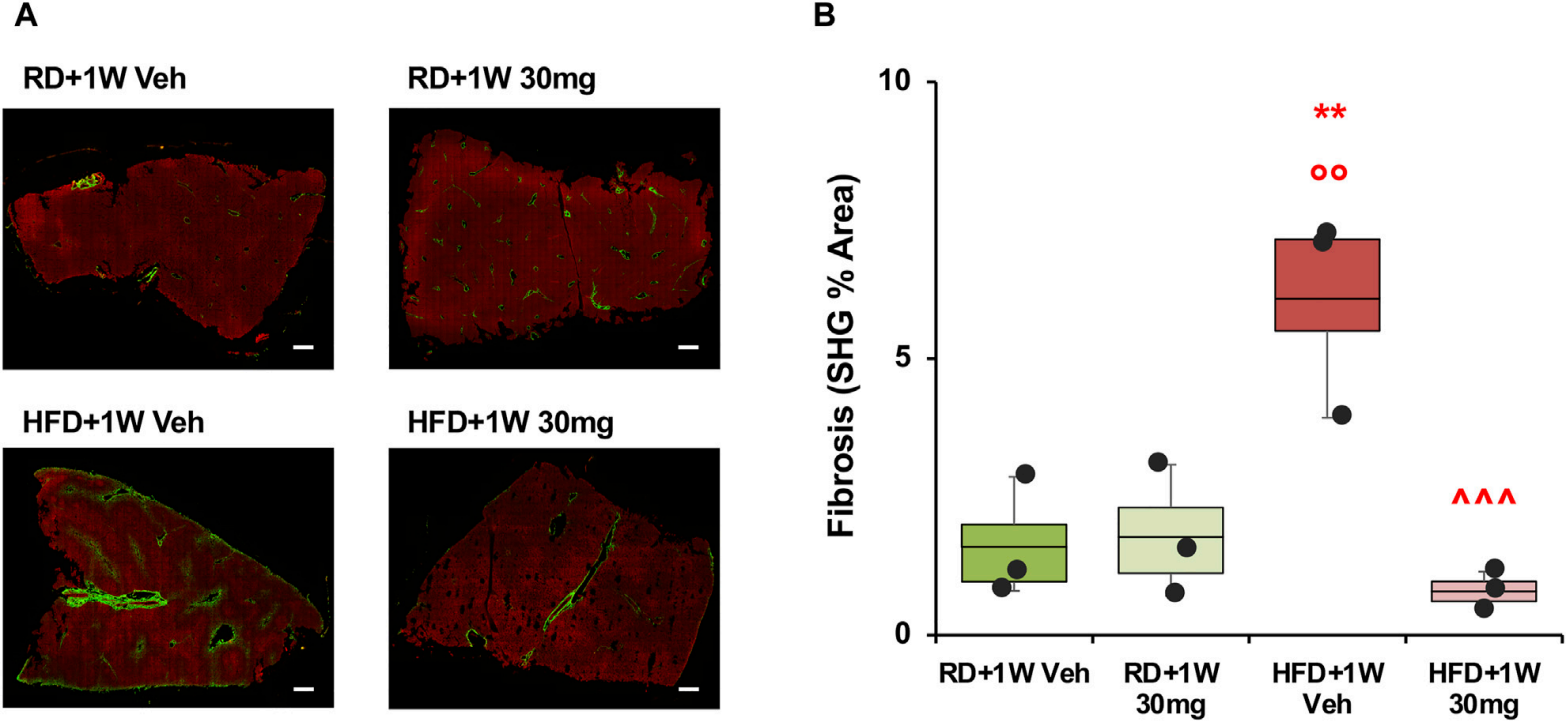
Second harmonic generation (SHG) analysis of collagen deposition in rabbit liver sections. Panel **(A)** shows representative images at ×20 original magnification of biopsies from each group (scale bar = 200 µm). Panel **(B)** shows quantification of the percentage of fibrosis area. Data are reported as box plots with data points (n = 3 in all groups). Significance was calculated using one-way parametric ANOVA test followed by *post hoc* Fisher’s Least Significant Difference (LSD) test for normally distributed data. **p < 0.01 vs. RD+1W Veh; °° *p* < 0.01 vs. RD+1W 30mg; ^^^ *p* < 0.001 vs. HFD+1W Veh.

Second harmonic generation (SHG) analyses confirmed the data observed with PSR staining, with a marked increase in HFD+1W Veh rabbits normalized by 1-week treatment with selvigaltin (Figures 4A,B; *p* < 0.001 vs. HFD + 1W Veh).

#### 3.1.4 Effects of acute selvigaltin treatment on MASLD-related liver mRNA gene expression

The mRNA expression of genes involved in the MASLD pathological processes was evaluated (Table 4). HFD induced an increase in several fibrosis and inflammation markers, as well as in the EDN1/TGFβ signaling pathway.

**TABLE 4:** Liver markers mRNA expression evaluated by RT-PCR.

EDN1/TGFβ pathway	RD+1W Veh	RD+1W 30 mg	HFD+1W Veh	Sign	HFD+1W 30 mg	Sign
*COL1A1*	*1.00 ± 0.62*	*0.82 ± 0.40*	*33.25 ± 17.86*	* °	*20.12 ± 15.24*	
**COL3A1**	**1.00 ± 0.12**	**0.47 ± 0.21**	**13.52 ± 6.89**	* °	**9.96 ± 7.51**	
**EDN1**	**1.00 ± 0.13**	**0.91 ± 0.20**	**1.51 ± 0.46**	**°**	**1.32 ± 0.21**	
**EDNRA**	**1.00 ± 0.16**	**0.89 ± 0.29**	**10.69 ± 7.22**	* °	**7.73 ± 5.69**	
**EDNRB**	**1.00 ± 0.19**	**0.79 ± 0.19**	**2.01 ± 0.29**	* °	**2.23 ± 0.95**	* °°
**αSMA**	**1.00 ± 0.28**	**0.96 ± 0.59**	**4.89 ± 2.70**		**5.77 ± 3.34**	* °
*SNAI2*	*1.00 ± 0.26*	*1.01 ± 0.31*	*2.54 ± 1.10*	* °	*1.55 ± 0.26*	
*TGFβ1*	*1.00 ± 0.16*	*0.95 ± 0.31*	*4.49 ± 1.45*	*°*	*4.25 ± 2.06*	*°*
*TGFβ3*	*1.00 ± 0.17*	*0.70 ± 0.08*	*3.03 ± 0.68*	*°*	*2.97 ± 1.96*	*°*

Fibrosis Markers	RD+1W Veh	RD+1W 30 mg	HFD+1W Veh	Sign	HFD+1W 30 mg	Sign
**FN1**	**1.00 ± 0.08**	**1.05 ± 0.12**	**1.19 ± 0.13**	*	**0.98 ± 0.14**	**°**
*IGF-1*	*1.00 ± 0.25*	*1.01 ± 0.19*	*0.42 ± 0.16*	*°*	*0.34 ± 0.08*	* °
*LGALS3*	*1.00 ± 0.38*	*1.27 ± 1.04*	*179.91 ± 24.72*	* °	*116.95 ± 77.56*	
**PAI-1**	**1.00 ± 0.88**	**2.63 ± 1.79**	**21.58 ± 2.45**	* °	**14.10 ± 16.47**	

Inflammation markers	RD+1W Veh	RD+1W 30 mg	HFD+1W Veh	Sign	HFD+1W 30 mg	Sign
*CD8* (*#*)	*1.00 ± 0.85*	*1.14 ± 0.81*	*2.01 ± 0.77*		*1.76 ± 0.20*	
**COX-2 (#)**	**1.00 ± 0.42**	**2.52 ± 1.59**	**19.23 ± 17.17**		**8.81 ± 10.30**	
**FOXP3**	**1.00 ± 0.19**	**0.86 ± 0.12**	**6.08 ± 4.01**	* °	**5.15 ± 2.78**	
**TBET (#)**	**1.00 ± 0.92**	**0.75 ± 0.20**	**1.38 ± 0.32**		**0.74 ± 0.14**	

Results are expressed as fold-change vs. RD+1W Veh and are reported as mean ± SD (n = 3 in all groups). Significance (Sign.): one-way parametric ANOVA, test followed by *post hoc* Fisher’s Least Significant Difference (LSD) test for normally distributed data (in bold) and one-way non-parametric ANOVA, Kruskal–Wallis test followed by *post hoc* Dunn’s analysis for not normally distributed data (in italic). No further test was performed when ANOVA, test resulted not significant (#). *p < 0.05 vs. RD+1W Veh; ° *p* < 0.05, °° *p* < 0.01 vs. RD+1W 30 mg.

Although selvigaltin numerically smoothed this increase, often the effect did not reach statistical significance (Table 4). In particular, after selvigaltin dosing in HFD rabbits, the significantly increased levels of COL1A1, COL3A1, EDN1, EDNRA, SNAI2, LGALS3, PAI-1, and FOXP3 mRNA expression were no longer statistically different from RD animals, while TBET showed a clear reduction trend compared to HFD untreated rabbits.

### 3.2 HFD rabbit study – prolonged, multiple dose selvigaltin treatment at weeks 9–12

#### 3.2.1 Selection of prolonged selvigaltin treatment doses and experimental groups

Based on results obtained with the acute (5-day) treatment of HFD rabbits with selvigaltin, we studied the effects of a longer (4-week) treatment, to evaluate potential therapeutic benefits of lower doses of the galectin-3 inhibitor selvigaltin.

Taking into consideration the increased plasma levels of selvigaltin observed during the acute treatment in HFD + 1W 30 mg animals, compared to the RD + 1W 30 mg group (Figure 1B), we selected lower doses of 0.3, 1.0, and 5.0 mg/kg/day for the 4-week *in vivo* treatment with selvigaltin. This allows a wider range of systemic selvigaltin concentrations to be tested to identify the level of free drug vs. galectin-3 affinity required for efficacy and to investigate PK/pharmacodynamic (PD) correlations. A subgroup of HFD rabbits were culled at 8 weeks, coinciding with the start of selvigaltin administration in other HFD groups, to evaluate the extent of metabolic and hepatic damages at treatment time 0 for comparison purposes.

Since we did not observe statistically significant differences in administering vehicle for 5 days or for 4 weeks neither in the RD nor in the HFD groups (data not shown), we pooled the respective groups in order to increase the number of available rabbits without unnecessarily increasing the number of animals sacrificed, in accordance with the Three Rs (Replacement, Reduction, Refinement) principle in animal experimentation (https://www.nc3rs.org.uk/; https://awionline.org/content/the-3rs).

#### 3.2.2 Selvigaltin pharmacokinetics

**FIGURE 5:**
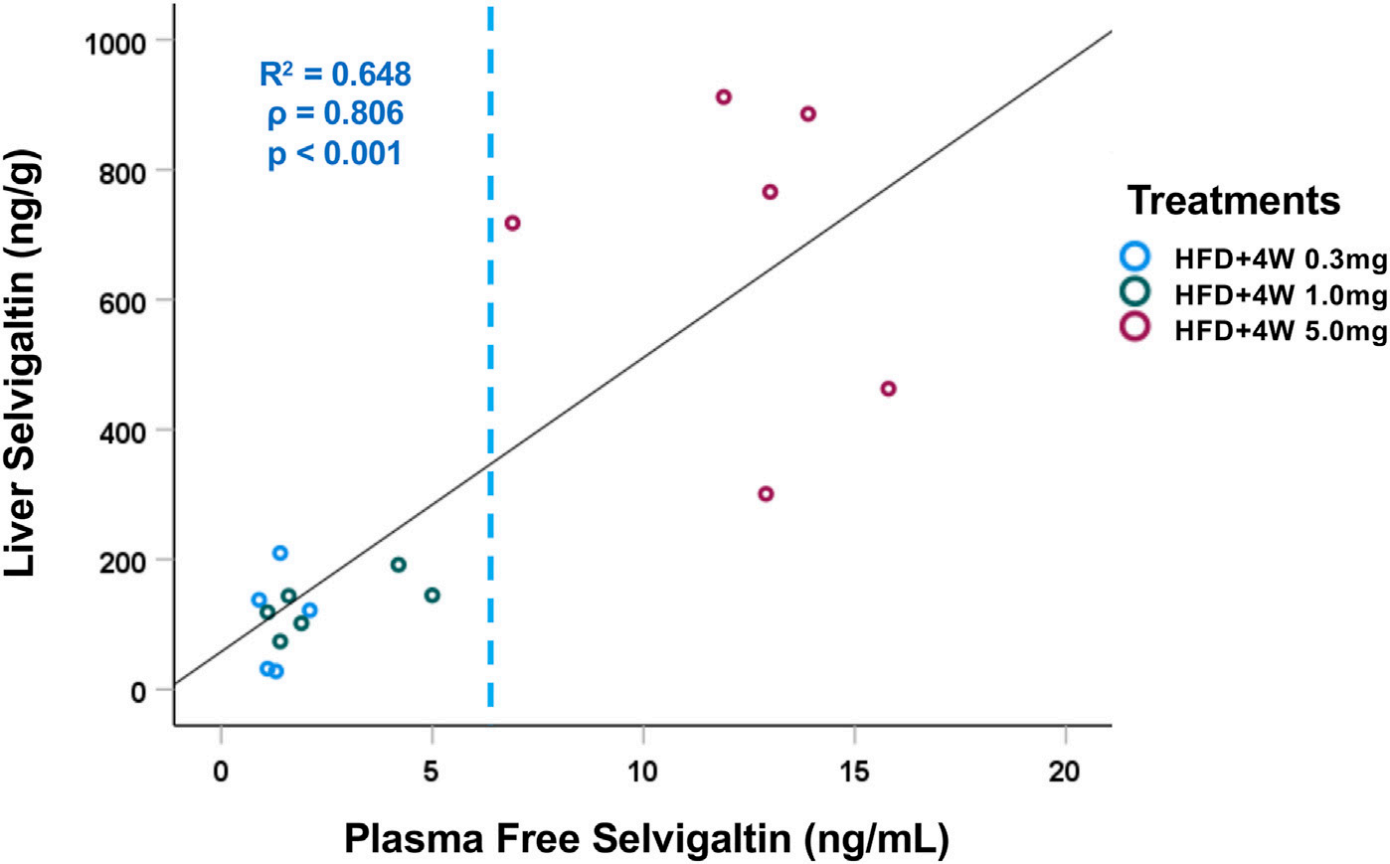
Correlation between plasma and liver tissue selvigaltin concentrations during treatment (HFD+4W 0.3mg, n = 5; HFD + 4W 1.0 mg, n = 6; HFD+4W 5.0 mg, n = 6). The dashed line indicates rabbit galectin-3 K_D_ (6.4 ng/mL). ρ = Spearman’s rank correlation coefficient; *R* ^2^ = square of Pearson correlation coefficient.

After 4 weeks of treatment with selvigaltin, the compound concentration was evaluated both in plasma and in liver tissue, observing a strong positive correlation (*p* < 0.001, Spearman’s test; Figure 5) with a 50–60-fold increased levels in the target organ. The concentration in either plasma or liver increased across increasing administered doses of selvigaltin.

#### 3.2.3 Effects of selvigaltin treatment on metabolic and biochemical parameters

Apart from a significant decrease in visceral fat induced by all the tested doses of selvigaltin, this treatment does not significantly alter most biochemical markers of MetS induced by HFD (Supplementary Table ST1).

However, a reduction of liver disease-related markers (γGT, AST, ALT, and bilirubin) was present in rabbits receiving selvigaltin, compared to the HFD group. In particular, AST, ALT, and bilirubin levels were significantly reduced by 1.0 mg selvigaltin treatment (all *p* < 0.05 vs. HFD + Veh). ALT levels were also significantly lowered by 0.3 mg and 5.0 mg dosing (*p* < 0.01 and *p* < 0.05 vs. HFD + Veh, respectively).

Bilirubin levels were evaluated weekly in all rabbits, starting at time 0 of selvigaltin administration, and a significant increase was observed in placebo-treated HFD rabbits only during the last week of treatment, when compared to selvigaltin-treated animals (Supplementary Figure SF1).

Several of the analyzed factors were already increased, compared to the RD groups, in rabbits sacrificed at 8 weeks, even though not reaching the levels observed in the 12-week HFD group (Supplementary Table ST1) and, interestingly, selvigaltin dosing induced an ALT reduction at or below the levels observed after 8 weeks of HFD.

#### 3.2.4 Effects of selvigaltin treatment on liver steatosis, galectin-3, and vimentin immunopositivity

**FIGURE 6:**
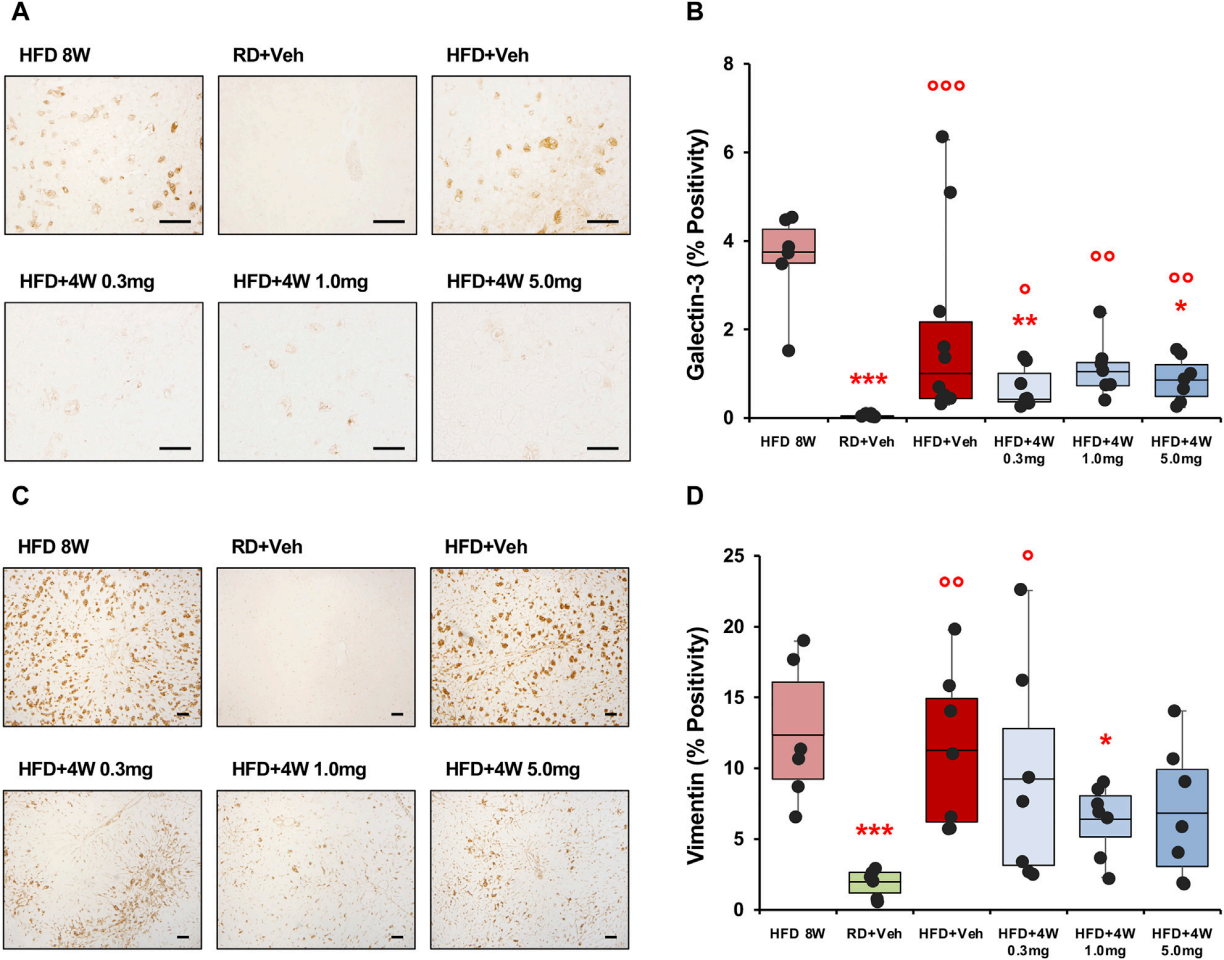
Galectin-3 and vimentin immunohistochemical analysis of liver sections. Panel **(A)** shows × 200 original magnifications of galectin-3 representative images (scale bar = 50 µm). Panel **(B)** shows the percentage of galectin-3 positivity (HFD 8W, n = 6; RD + Veh, n = 9; HFD + Veh, n = 10; HFD + 4W 0.3 mg, n = 7; HFD + 4W 1.0 mg, n = 7; HFD + 4W 5.0 mg, n = 7). Panel **(C)** shows ×40 original magnifications of vimentin representative images (scale bar = 100 µm). Panel **(D)** shows quantification of vimentin positivity (HFD 8W, n = 6; RD + Veh, n = 6; HFD + Veh, n = 7; HFD + 4W 0.3 mg, n = 7; HFD + 4W 1.0 mg, n = 7; HFD + 4W 5.0 mg, n = 7). Data are reported as box plots with data points. Significance was calculated using one-way non-parametric ANOVA Kruskal–Wallis test followed by *post hoc* Dunn’s analysis for not normally distributed data (Panel B) and one-way parametric ANOVA test followed by *post hoc* Fisher’s Least Significant Difference (LSD) test for normally distributed data (Panel D). *p < 0.05, **p < 0.01, ***p < 0.001 vs. HFD 8W; ° *p* < 0.05, °° *p* < 0.01, °°° *p* < 0.001 vs. RD + Veh.

Liver steatosis was evaluated, as previously, by analyzing liver specimens from all experimental groups with Oil Red O staining (Supplementary Figure SF2). The presence of steatosis was confirmed in all HFD groups, including the 8-week and selvigaltin-treated rabbits, without significant differences among HFD groups, eventhough a mild beneficial effect could be observed in the 1.0 mg treatment group, which was not significantly different from controls. Galectin-3 staining and immunolocalization in liver was evaluated by analyzing liver specimens from all experimental groups, with the significant increase evidenced in HFD rabbits only partially abated by all selvigaltin treatments (Figures 6A,B).

We then evaluated vimentin positivity and immunolocalization by analyzing liver specimens from all experimental rabbits. Figure 6C shows ×40 original magnification representative images from liver specimens. The positivity area is reported in Figure 6D and, similarly to galectin-3 data, the higher vimentin percentages were observed in the 8-week HFD and placebo-treated HFD groups, whereas 1.0 mg selvigaltin treatment induced a borderline reduction in vimentin positivity (*p* = 0.072 vs. HFD + Veh).

Data collected from galectin-3 and vimentin positivity were analyzed as bivariate with Spearman’s test, and a highly significant correlation was observed (*p* < 0.01; Supplementary Figure SF3).

#### 3.2.5 Galectin-3 colocalizes with vimentin and RAM11

**FIGURE 7:**
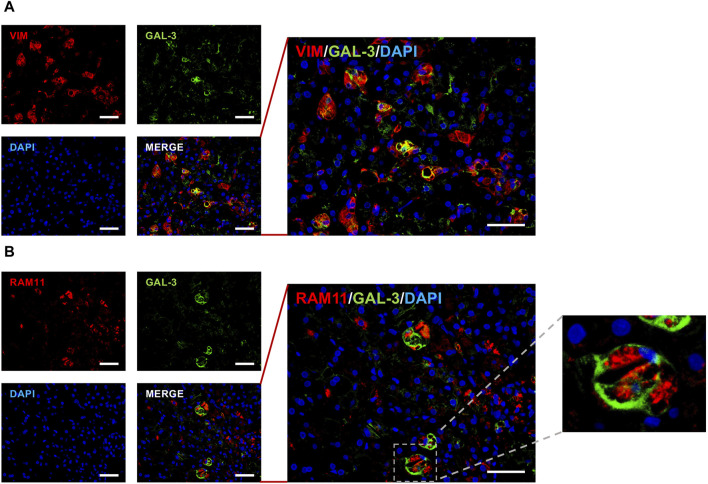
Galectin-3, vimentin and RAM11 staining. Representative dual immunofluorescent staining of galectin-3 (green color) with either vimentin red color, panel **(A)** or RAM11 red color, panel **(B)** in HFD + Veh hepatic slides (n = 3). Corresponding nuclear DAPI labeling is also reported (blue color) in both panels. The quantitative analysis was performed by counting positive cells in at least 6 fields per slide. ×200 original magnifications; scale bar = 50 µm.

The previous result prompted us to evaluate the possible colocalization of galectin-3 and vimentin, with images of hepatic slides reported in Figure 7A clearly showing a partial colocalization of the two molecules. The enlargement of the merged data, shown on the right of panel A, confirms this observation.

Since vimentin, beside a stellate cells marker, is also an activated macrophage marker, we analyzed the putative colocalization of galectin-3 with RAM11 (cytoplasmatic antigen of rabbit macrophages), and results are reported in Figure 7B.

The images show that galectin-3/RAM11 positive cells are 36.5% ± 3.6% of RAM11-positive macrophages, as highlighted by the merged data enlargement in the middle of panel B. Immunofluorescent morphoanalysis showed RAM11 cytoplasmatic staining and cell surface galectin-3 expression, typical of circulating monocyte-derived macrophages (Figure 7B, further enlargement on the right).

#### 3.2.6 Histological effects of selvigaltin treatment on liver inflammation, hepatocyte ballooning and fibrosis

Taking into consideration the potential involvement of macrophages, and therefore pro-inflammatory processes, we then analyzed, as we did for the 5-days acute treatment study, the selvigaltin effects on inflammation (Giemsa staining; Supplementary Figure SF4, panel A; Supplementary Table ST2), as well as on hepatocyte ballooning (Masson’s trichrome staining; Supplementary Figure SF4, panel B; Supplementary Table ST2) and on fibrosis Ishak scores (PSR staining; ×100 original magnifications; Supplementary Figure SF4, panel C; Supplementary Table ST2).

Prominent inflammation foci were evident in the 8-week HFD group, in the placebo HFD group, and in the 0.3 mg selvigaltin group (Supplementary Figure SF4, panel A, arrows), and this feature was partially reduced by both 1.0 mg and 5.0 mg selvigaltin treatments. Similarly, hepatocyte ballooning was mainly observed in the HFD and 0.3 mg selvigaltin groups, with higher doses of the galectin-3 inhibitor reducing Masson’s trichrome staining (Supplementary Figure SF4, panel B).

Interestingly, the extensive fibrotic depositions observed in the HFD placebo-treated rabbits, especially in terms of perisinusoidal fibers, were markedly reduced by selvigaltin (1.0 mg and 5.0 mg; Supplementary Figure SF4, panel C), notably with statistically significant reduction of the Ishak score in the 1.0 mg selvigaltin group (*p* < 0.05 vs. HFD + Veh), as reported in the contingency table (Supplementary Table ST2), which confirms the beneficial effects of both 1.0 mg and 5.0 mg selvigaltin treatments on these variables.

**FIGURE 8:**
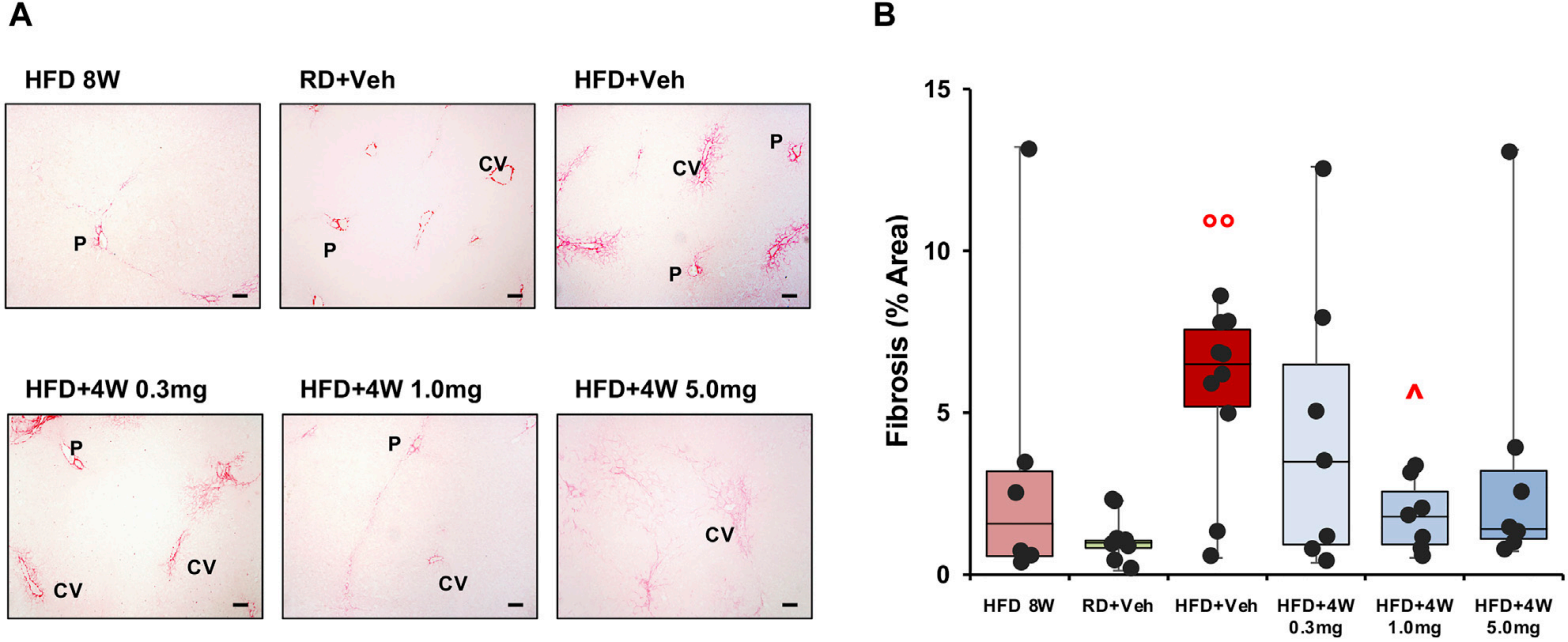
Picrosirius Red analysis of collagen deposition in rabbit liver sections. Panel **(A)** shows representative images of ×40 original magnification slides (scale bar = 100 µm). P, portal vein; CV, centrilobular vein. Panel **(B)** shows the percentage of fibrosis area in rabbit liver biopsies. Data are reported as box plots with data points (HFD 8W, n = 6; RD + Veh, n = 9; HFD + Veh, n = 10; HFD 4W 0.3 mg, n = 7; HFD + 4W 1.0 mg, n = 7; HFD + 4W 5.0 mg, n = 7). Significance was calculated using one-way non-parametric ANOVA Kruskal–Wallis test followed by *post hoc* Dunn’s analysis for not normally distributed data. °° *p* < 0.01 vs. RD + Veh; ^ *p* < 0.05 vs. HFD + Veh.

Figure 8A shows ×40 original magnifications representative images of liver biopsies PSR staining. Figure 8B bar graph reports the mean densitometry of the collagen deposition in the region of interest (ROI) sampled areas of all animals. Whereas at 8 weeks the fibrotic process is beginning to emerge, the HFD + Veh data shows a full-blown fibrosis (*p* < 0.01 vs. RD + Veh), only partially counteracted by the 0.3 mg and 5.0 mg selvigaltin dosages but normalized by the 1.0 mg selvigaltin dose (*p* < 0.05 vs. HFD + Veh).

#### 3.2.7 Effects of selvigaltin treatment on fibrosis and inflammation markers gene expression

The bivariate Spearman’s correlation analysis was applied between PSR area and mRNA expression of markers relevant to the TGFβ cascade, finding a highly significant correlation of PSR area with TGFβ3 (*p* < 0.01), SNAI2 (*p* = 0.001), COL1A1 (*p* < 0.001), and COL3A1 (*p* < 0.001) (Supplementary Figure SF5).

We therefore conducted a wider mRNA expression analysis of several markers linked to either fibrosis or inflammation. Supplementary Table ST3 shows the results obtained in all experimental groups. Several fibrosis markers, including LGALS3, significantly increased in HFD + Veh animals, were significantly reduced or normalized by 4-week selvigaltin 1.0 mg treatment, a result partially mirrored by the 0.3 mg and the 5.0 mg selvigaltin treatment.

Likewise, the selvigaltin 1.0 mg treatment appeared to be the most effective in reducing the HFD-induced increase of several inflammation markers, particularly the mRNA expression of IL6, one the initiators of the inflammation-to-fibrosis cascade. The Spearman’s bivariate analyses showed a highly significant correlation of LGALS3 with TGFβ3, SNAI2, COL1A1, and COL3A1 (all *p* < 0.001; Supplementary Figure SF6), further confirming the pivotal role of galectin-3 in hepatic fibrosis.

#### 3.2.8 Effects of selvigaltin treatment on fibrosis and inflammation markers protein expression

To confirm the pro-fibrosis and pro-inflammatory mRNA markers data at protein level, we evaluated collagen, IL6 and TNFα protein contents in the liver homogenates. Supplementary Table ST4 shows the results obtained in all experimental groups. Liver collagen significantly increased in the HFD + Veh group (*p* < 0.001 vs. RD + Veh), with both 1.0 mg and 5.0 mg selvigaltin treatments reducing it significantly (*p* < 0.001 and *p* < 0.01, respectively). Likewise, pro-inflammatory markers IL6 and TNFα protein expression in liver homogenized biopsies showed either a trend (IL6: *p* = 0.144 vs. HFD + Veh with 1.0 mg and 5.0 mg selvigaltin treatments) or a statistically significant reduction (TNFα: *p* < 0.05 vs. HFD + Veh with 1.0 mg and 5.0 mg selvigaltin treatments) compared to the observed HFD-induced significant increase (IL6 and TNFα, both *p* < 0.001 vs. RD + Veh).

#### 3.2.9 Detailed fibrosis evaluation and endpoint comparison analysis

When data collected from PSR fibrosis areas and SHG variables are analyzed as bivariate with Spearman’s test, a highly significant correlation was observed for PSR area percentage with SHG overall area (*p* < 0.01), strings overall length (*p* < 0.001), length of portal tract strings (*p* < 0.001), and length of perisinusoidal region strings (*p* = 0.001) (Supplementary Figure SF7).

**FIGURE 9:**
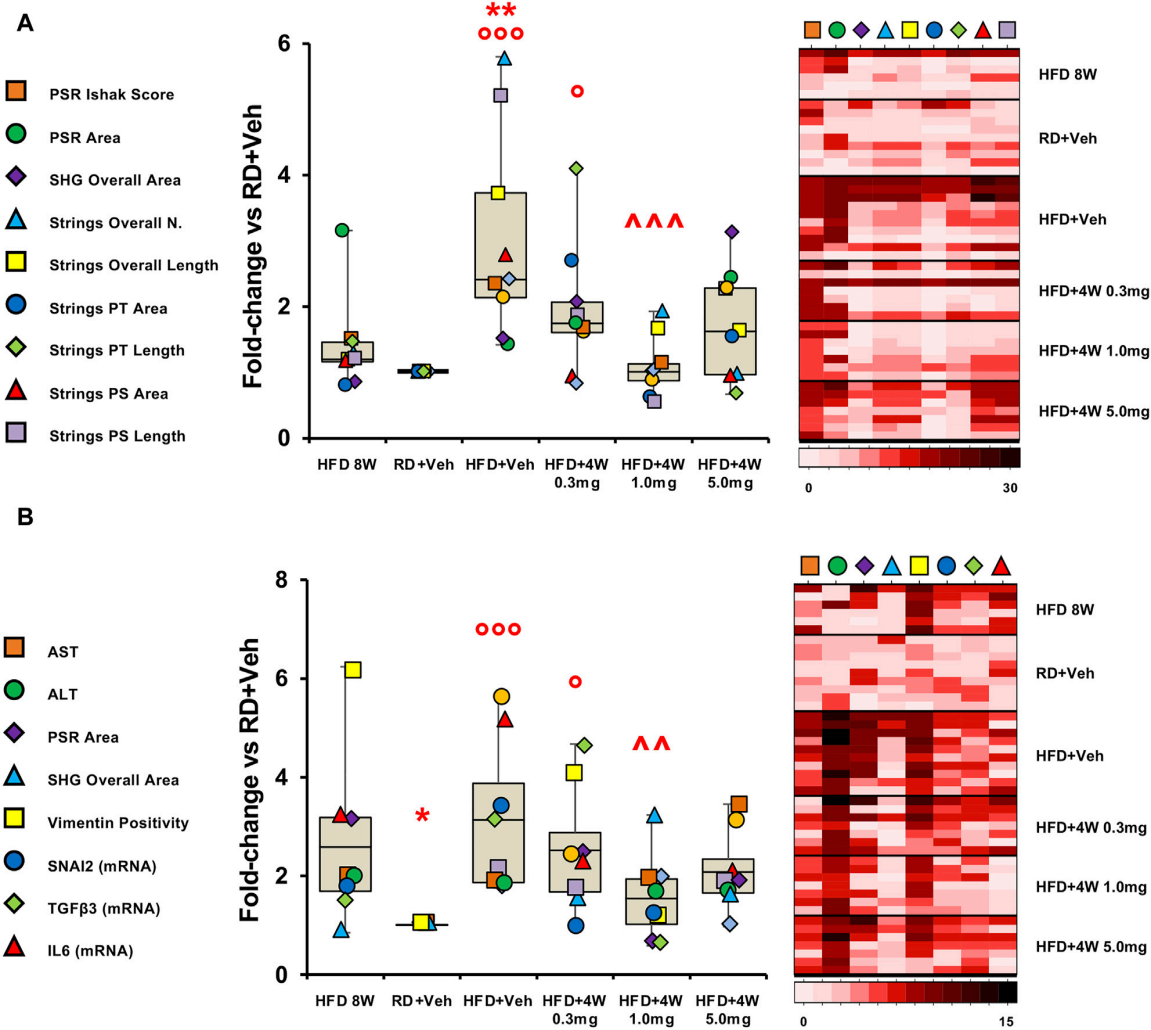
Fibrosis evaluation and endpoint comparison. Panel **(A)** shows fibrosis markers graph and heatmap in rabbit liver sections. Panel **(B)** shows liver damage and pro-fibrotic markers graph and heatmap in rabbit liver sections and homogenates. Each variable is expressed as fold-change mean vs. RD + Veh and represented by a different symbol. Data are reported as box plots with data points (HFD 8W, n = 6; RD + Veh, n = 9; HFD + Veh, n = 10; HFD + 4W 0.3 mg, n = 7; HFD + 4W 1.0 mg, n = 7; HFD + 4W 5.0 mg, n = 7). Significance was calculated using one-way non-parametric ANOVA Kruskal–Wallis test followed by *post hoc* Dunn’s analysis for not normally distributed data (Panel A) and one-way parametric ANOVA test followed by *post hoc* Fisher’s Least Significant Difference (LSD) test for normally distributed data (Panel B). *p < 0.05, **p < 0.01 vs. HFD 8W; ° *p* < 0.05, °°° *p* < 0.001 vs. RD + Veh; ^^ *p* < 0.01, ^^^ *p* < 0.001 vs. HFD + Veh. Heatmap representation of data for each group is showed on the right. PSR, picrosirius red; SHG, second harmonic generation; PT, portal tract; PS, perisinusoidal region.

The different methods of evaluating fibrosis (Ishak score, PSR and SHG) were therefore compared in the experimental groups, expressing results as fold-changes vs. the RD group, and assessing the mean of the fold-change for each variable in the different experimental groups. Results are reported as graph and heatmap in Figure 9A.

Remarkably, all three selvigaltin doses were able to reduce mean liver fibrosis, as assessed by fold-change for each variable, compared to HFD + Veh animals, reaching statistical significance for the 1.0 mg selvigaltin dosing (*p* < 0.001 vs. HFD + Veh), and showing a nearly significant trend for the other treatments (*p* = 0.137 for 0.3 mg and *p* = 0.059 for 5.0 mg, both vs. HFD + Veh). Of note, data at 8 weeks suggest that fibrosis is either absent or at an early stage. The heatmap (right panel in Figure 9A) provides a visual representation, confirming these data.

A clear pattern appeared when the full set of results was taken into consideration, with data converging towards an IL6/TGFβ-driven pathway responsible for the hepatic fibrosis development. Accordingly, the most relevant findings in this study have been assembled in graph bars (Figure 9B). Variables include circulating markers of liver damage (AST, ALT), immunohistochemistry analyses of pro-fibrogenic factors (vimentin, PSR, SHG), and mRNA expression of key markers (IL6, TGFβ3, SNAI2).

These data, reported as fold-changes vs. the RD group and comparing the mean of the fold-changes for each variable considered, are summarized as graph and heatmap in Figure 9B. Overall, both 1.0 mg and 5.0 mg selvigaltin treatment groups showed a significant or near significant positive effect, compared to HFD animals receiving placebo (*p* < 0.01 and *p* = 0.080, respectively), whereas the lower dose selvigaltin group (0.3 mg), although displaying a numerical decrease, resulted far from reaching statistical significance (*p* = 0.298).

## 4 Discussion

Inhibition of galectin-3 in liver disease is hypothesized to offer therapeutic potential (Mackinnon et al., 2023), with early clinical studies starting to demonstrate effects on biomarkers of liver injury (Lindmark et al., 2023). In this study we aimed to further investigate the efficacy of selvigaltin, the most clinically advanced galectin-3 oral small molecule inhibitor, in an HFD-induced MetS rabbit model to provide insight into predicted pharmacologically active doses in human. Studies in rabbits have the potential to be more informative and translational to humans compared to mice as a result of closer Carbohydrate Recognition Domain binding site sequence homology, and therefore selvigaltin affinity, between rabbit and human galectin-3. The first part of the study was aimed at ascertaining tolerability, PK and effects on liver fibrosis of selvigaltin, when a high pharmacological dose [30 mg/kg/day with human equivalent dose (HED) of −580 mg based on a 60 kg adult and applying the guidance provided by the US Food Drug Administration (FDA Guidance Document, 2005)] was administered during the last week in a 12-week HFD-induced MetS rabbit model.

This high dose of selvigaltin was well tolerated and this enabled its PK profile to be defined over the course of dosing. The steady state systemic plasma drug concentration was ∼40-fold greater than the selvigaltin rabbit galectin-3 binding K_D_, allowing improved modelling of future doses. Untreated male rabbits fed an HFD for 12 weeks showed all the hallmarks of a clinically developed MetS, confirming the validity and reproducibility of the model, as previously reported (Filippi et al., 2009; Maneschi et al., 2013; Vignozzi et al., 2014; Comeglio et al., 2018). The acute treatment with selvigaltin in HFD animals showed a significant reduction in cholesterol, triglycerides, and ALT circulating levels, compared to placebo-treated HFD rabbits. HFD animals treated with selvigaltin also showed a trend in the reduction of established key fibrogenic transcripts and of a variety of inflammation markers. Although the effects did not always reach significance, likely due to the reduced animal numbers in this enabling study, the trends stood as an encouraging sign supporting the investigation of selvigaltin in a chronic dosing study. In addition, the elevated levels of galectin-3 and related mechanistic biomarkers of fibrosis in the liver from RD to HFD animals also provided further validation of the model as suitable for measuring galectin-3 inhibition. Finally, selvigaltin 1-week treatment appeared beneficial in reducing liver inflammation, ballooning, and, more importantly, normalizing the HFD-induced fibrosis, as assessed by different histochemistry methods.

The positive effect of the single 30 mg/kg/day dose across a range of biomarkers following acute dosing, most importantly on reversing HFD-induced fibrosis, supported further investigation adopting a longer dosing period of selvigaltin over a lower dosing range. In order to allow a relevant human equivalent dose (HED) to be covered in the chronic study [current clinic studies with selvigaltin dose 100 mg twice a day (Lindmark et al., 2023)], 0.3 mg/kg/day, 1 mg/kg/day and 5 mg/kg/day selvigaltin doses were selected in a prolonged 4-week *in vivo* treatment. These doses were predicted to result in a range of systemic concentrations, based on a 30 mg/kg/day steady state dose, which would cover multiples below and above the galectin-3 rabbit K_D_ and human equivalent doses (HEDs) of −6 mg, 20 mg, and 100 mg. This allows a crude estimate for the exposure required systemically in humans of selvigaltin to achieve efficacy by extrapolating PK data and affinity in humans (Zetterberg et al., 2022; Aslanis et al., 2023; Lindmark et al., 2023). In addition, this would also enable a more thorough investigation of PK/PD relationships between drug levels and biomarkers perturbed by selvigaltin in the HFD rabbits over longer dosing.

Terminal PK in the chronic dosing study confirmed an expected range of concentrations for unbound selvigaltin that covered levels above and below the rabbit K_D_. There was a linear increase across the ascending dose groups which correlated with elevated total drug levels in the liver. Although the 4-week dosing study of selvigaltin did not provide significant benefits for most MetS biomarkers, the significant excess of abdominal visceral fat observed in HFD placebo rabbits, a major risk factor for the development of metabolic syndrome (Després, 2006; Després and Lemieux, 2006), was drastically reduced in the selvigaltin-treated HFD groups, albeit without a corresponding significant reduction in the liver steatosis. The treatment beneficial effect on fibrosis not accompanied by a significant reduction of steatosis could be possibly explained by a slower clearance of hepatic fat infarction, due to several co-intervening factors, including but not limited to a lack of change in lifestyle habits of HFD animals, which albeit treated do not change diet or exercise routine, and also to a relatively short treatment time. Indeed, we are beginning to observe a reduction in hepatic steatosis in the 1.0 mg group, and there is an effect of the treatment on visceral fat deposition. It was beyond the remit of this paper to identify visceral fat as a putative target organ for selvigaltin, but a longer protocol and treatment regime might address this point. Selvigaltin 4-week administration also induced a decrease of liver damage circulating factors, in particular transaminases and bilirubin. Previous studies demonstrated that bilirubin levels are increased in animal models receiving HFD (Arias-Mutis et al., 2017; Gabbia et al., 2019), mainly due to bile ducts system obstruction, and bilirubin is a marker of liver damage (Harmon et al., 2011) (https://www.mayoclinic.org). Interestingly, bilirubin circulating levels, evaluated weekly in all groups from week 8–12, showed a significant increase in placebo-treated HFDs during the last week of treatment, a possible indication of a late-onset severe liver disease. At 8 weeks, the HFD animals displayed evidence of an active inflammation process and milder evidence of ballooning and fibrosis, with the former subsidizing and the latter worsening at 12 weeks, recapitulating the clinical evidence. Indeed, the 8-week HFD rabbits showed several factors indicating an already developing metabolic syndrome process with, crucially, strong liver immunopositivity for inflammation, galectin-3 and vimentin, a marker for stellate cells (Puche et al., 2013) and activated macrophages (Mor-Vaknin et al., 2003), even though with limited involvement of biochemical hepatic markers. This might translate into inflammation-driven initiation of the fibrotic process, a hypothesis corroborated by the colocalization of galectin-3 and rabbit macrophage marker RAM11 we observed in 12-week HFD liver biopsies. Indeed, galectin-3 has been previously found localized on the surface of macrophages derived from circulating monocytes (Liu et al., 1995; Kram, 2023). Treatment with 1.0 mg or 5.0 mg selvigaltin induced an amelioration in terms of inflammation, ballooning and fibrosis, the latter particularly evident at perisinusoidal/portal tract level and showing a strong correlation among different methods of evaluation.

These results were confirmed at the molecular level, firstly observing a strong Spearman’s correlation for PSR data and fibrotic markers mRNA expression (TGFβ, SNAI, collagens), which, in turn correlated with galectin-3 mRNA expression, also reduced by 1.0 mg and 5.0 mg selvigaltin treatments. Fibrogenic markers like TGFβ3 and SNAI2 are paramount for remodeling and cell-matrix adhesion, thus contributing to the epithelial-to-mesenchymal cell transition (EMT) (Thiery and Sleeman, 2006). Interestingly, it has been reported how galectin-3 can amplify TGFβ signaling by forming lattices on cell surfaces, thus allowing TGFβ receptor entrapment and prolonged action (Nabi et al., 2015). Moreover, the selvigaltin beneficial effect was also observed on the decreased mRNA expression of IL6, a cytokine strongly connected to a compromised tissue repair and the shifting from acute inflammation into a more chronic profibrotic state (Fielding et al., 2014; Li et al., 2022). The converging data of HFD effects on biochemical circulating measures of liver injury (transaminases), inflammation, molecular analyses and fibrosis bring forward the paradigm of an IL6/TGFβ-induced pathway responsible for hepatic collagen deposition, with selvigaltin dose-dependently and significantly improving all aspects. Unfortunately, as only one of the doses administered (0.3 mg) was shown to have a partial effect across the endpoints measured, it was not possible to build a strong PK/PD relationship for individual biomarkers. This suggests lower doses may be required to enrich this dataset and further enhance the analysis of biomarkers and their PK/PD relationship for translation into clinic studies.

The Food and Drug Administration recommended clinical MASH (formerly classified as NASH) endpoints guidance for treatments (FDA Guidance Document, 2018) states that the reasonable predictions of clinical benefit are 1) resolution of steatohepatitis on overall histopathological reading and no worsening of liver fibrosis; OR 2) improvement in liver fibrosis and no worsening of steatohepatitis; OR 3) both resolution of steatohepatitis and improvement of fibrosis. With respect to the above recommended clinical MASH endpoints, selvigaltin 1.0 mg and 5.0 mg treatments significantly reduced steatohepatitis (inflammation, ballooning) and hepatic fibrosis, compared to placebo-treated HFD rabbits. Providing additional evidence for MASH improvement, molecular data showed reduction of major proinflammatory and profibrotic markers.

In conclusion, the preclinical findings following selvigaltin treatment in an experimental HFD-induced rabbit model of MASH and fibrosis support the human selvigaltin dose of 100 mg twice daily currently being investigated in clinical studies of liver cirrhosis (Lindmark et al., 2023) (ClinicTrials.gov Identifier: NCT05009680, 2023). The data in the model also suggest that once a day dosing of lower doses than 100 mg in humans could be efficacious and are worthy of investigation in future clinic studies. From a clinical point of view, the most important results are those described in the first part of the present study, showing a rapid therapeutic effect of selvigaltin in already established MASH. Most of the patients, indeed, require therapy only when liver inflammation and fibrosis are already present. Notably, the second part of this study suggests that even lower dosages can be effective. However, lower selvigaltin dosages should be assessed in advanced stages of the MASH, to replicate the clinal scenario. The present study clearly shows, for the first time, that using a simplistic method for predicting efficacy based on systemic unbound drug levels vs. an *in vitro* measure of affinity or potency is not appropriate for galectin-3 as a drug target. Levels of unbound selvigaltin below galectin-3 K_D_ have demonstrated significant efficacy on a range of biomarkers of MASH and fibrosis, showing that high concentrations in excess of K_D_ are not required for activity. This is perhaps not surprising, considering the pleiotropic nature of galectin-3 interacting with multiple receptors and mechanisms rather than a single receptor mechanism. In addition, this study adds further to the validation of galectin-3 as a therapeutic target in liver disease, supporting the ongoing clinical development of selvigaltin.

## Figures and Tables

**Table:** 

**ALT**	alanine aminotransferase
**AST**	aspartate aminotransferase
**AUC**	area under curve
**BW**	body weight
**CD4**	cluster of differentiation 4
**CD8**	cluster of differentiation 8
**COL1A1**	collagen type I alpha 1
**COL3A1**	collagen type III alpha 1
**COX-2**	inducible cyclooxygenase-2
**CRD**	carbohydrate recognition domain
**EDN1**	endothelin 1
**EDNRA**	endothelin receptor type A
**EDNRB**	endothelin receptor type B
**EMT**	epithelial-mesenchymal-transition
**FN1**	fibronectin 1
**FOXP3**	forkhead box P3
**FP**	fluorescence polarization
**γGT**	gamma-glutamyltransferase
**IGF-1**	insulin-like growth factor 1
**H&E**	hematoxylin and eosin
**HED**	human equivalent dose
**HFD**	high fat diet
**IL1β**	interleukin-1, subunit beta
**IL2**	interleukin-2
**IL6**	interleukin-6
**LGALS3**	galectin-3
**LOD**	limit of detection
**LOQ**	limit of quantification
**MAP**	mean arterial blood pressure
**MASH**	metabolic-associated steatohepatitis
**MASLD**	metabolic dysfunction-associated steatotic liver disease
**MCP1**	monocyte chemoattractant protein-1
**MetS**	metabolic syndrome
**NAFLD**	non-alcoholic fatty liver disease
**NASH**	nonalcoholic steatohepatitis
**RD**	regular diet
**OGTT**	oral glucose tolerance test
**PAI-1**	plasminogen activator inhibitor-1
**PD**	pharmacodynamic
**PK**	pharmacokinetics
**PS**	perisinusoidal region
**PSR**	picrosirius red
**PT**	portal tract
**RAGE**	receptor for advanced glycation endproducts
**ROI**	region of interest
**SD**	standard deviation
**SHG**	second harmonic generation
**αSMA**	alpha smooth muscle actin
**SNAI1**	snail family transcriptional repressor 1
**SNAI2**	snail family transcriptional repressor 2
**TBET**	T-box transcription factor TBX21
**TPEF**	two-photon excitation fluorescence
**TGFβ1**	transforming growth factor beta 1
**TGFβ3**	transforming growth factor beta 3
**TLR4**	toll-like receptor 4
**TNFα**	tumor necrosis factor alpha
**VAT**	visceral adipose tissue

## Data Availability

The original contributions presented in the study are included in the article/Supplementary Material, further inquiries can be directed to the corresponding author.
