# Supplementary figures and images for: The galectin-3 inhibitor selvigaltin reduces liver inflammation and fibrosis in a high fat diet rabbit model of metabolic-associated steatohepatitis
Source: Front Pharmacol. 2024 Jul 31;15:1430109. doi: 10.3389/fphar.2024.1430109 (PMC11322497; doi:10.3389/fphar.2024.1430109)

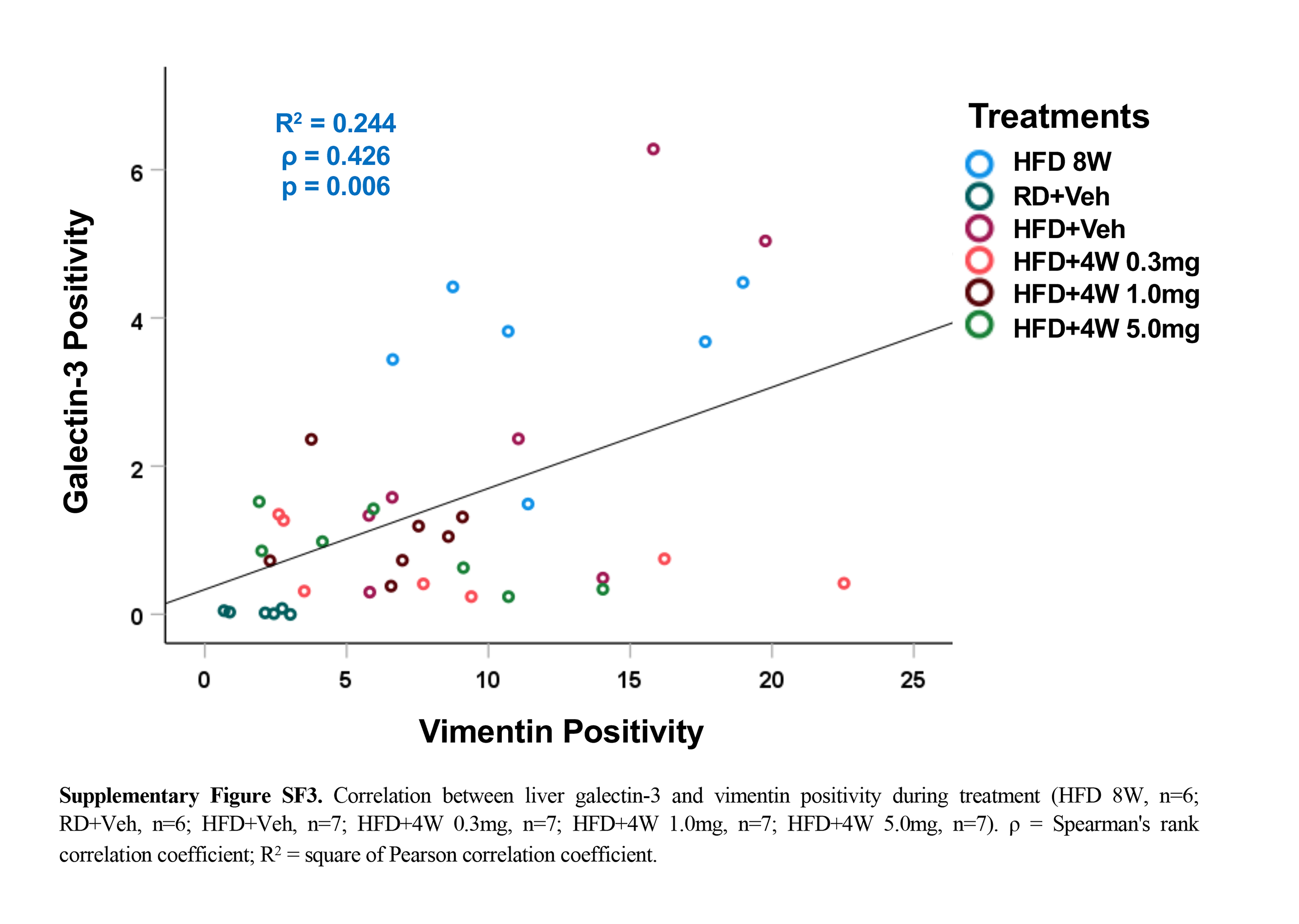

Supplement: Supplementary file 1 [file Image3.JPEG]

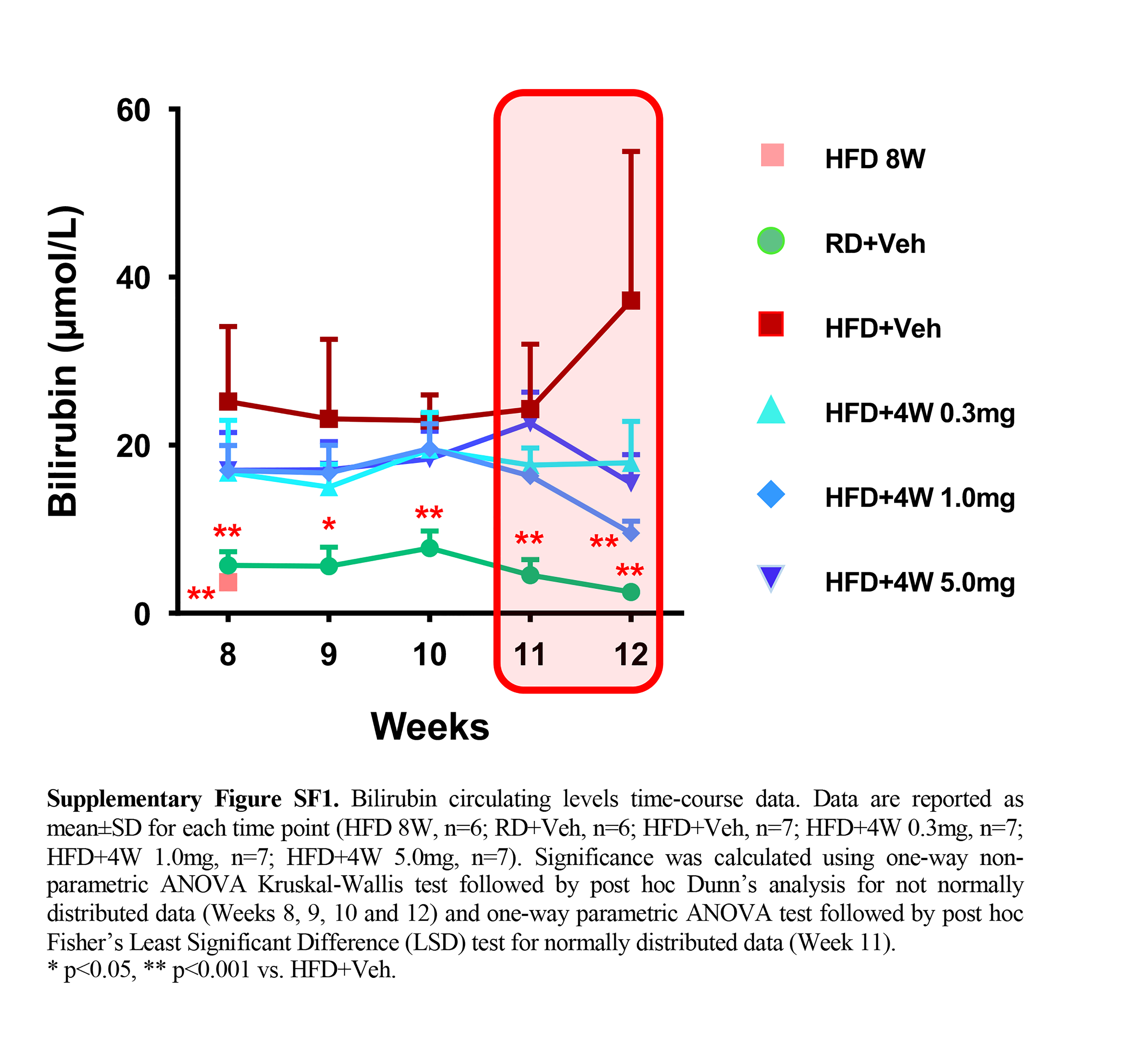

Supplement: Supplementary file 3 [file Image1.JPEG]

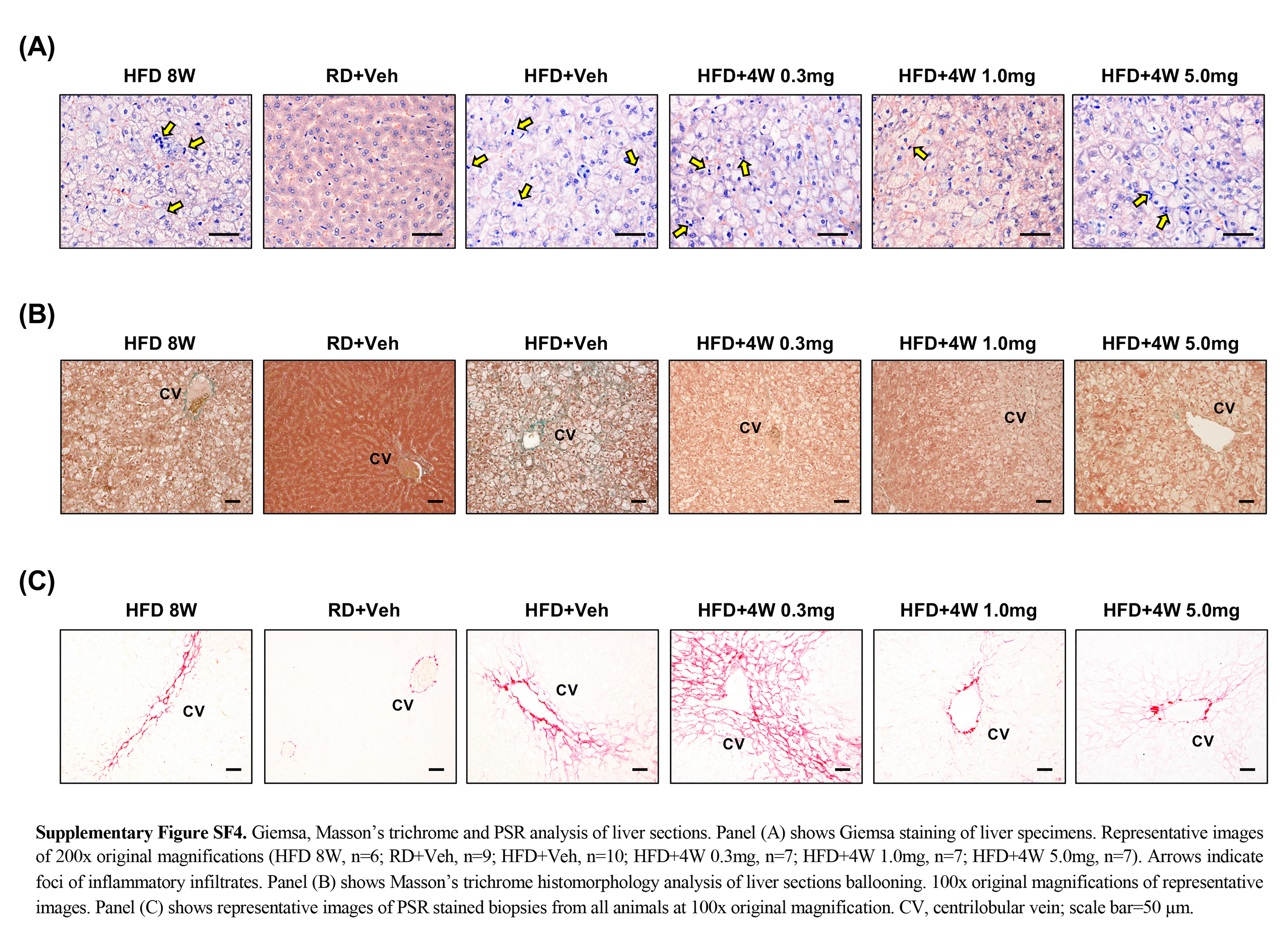

Supplement: Supplementary file 4 [file Image4.JPEG]

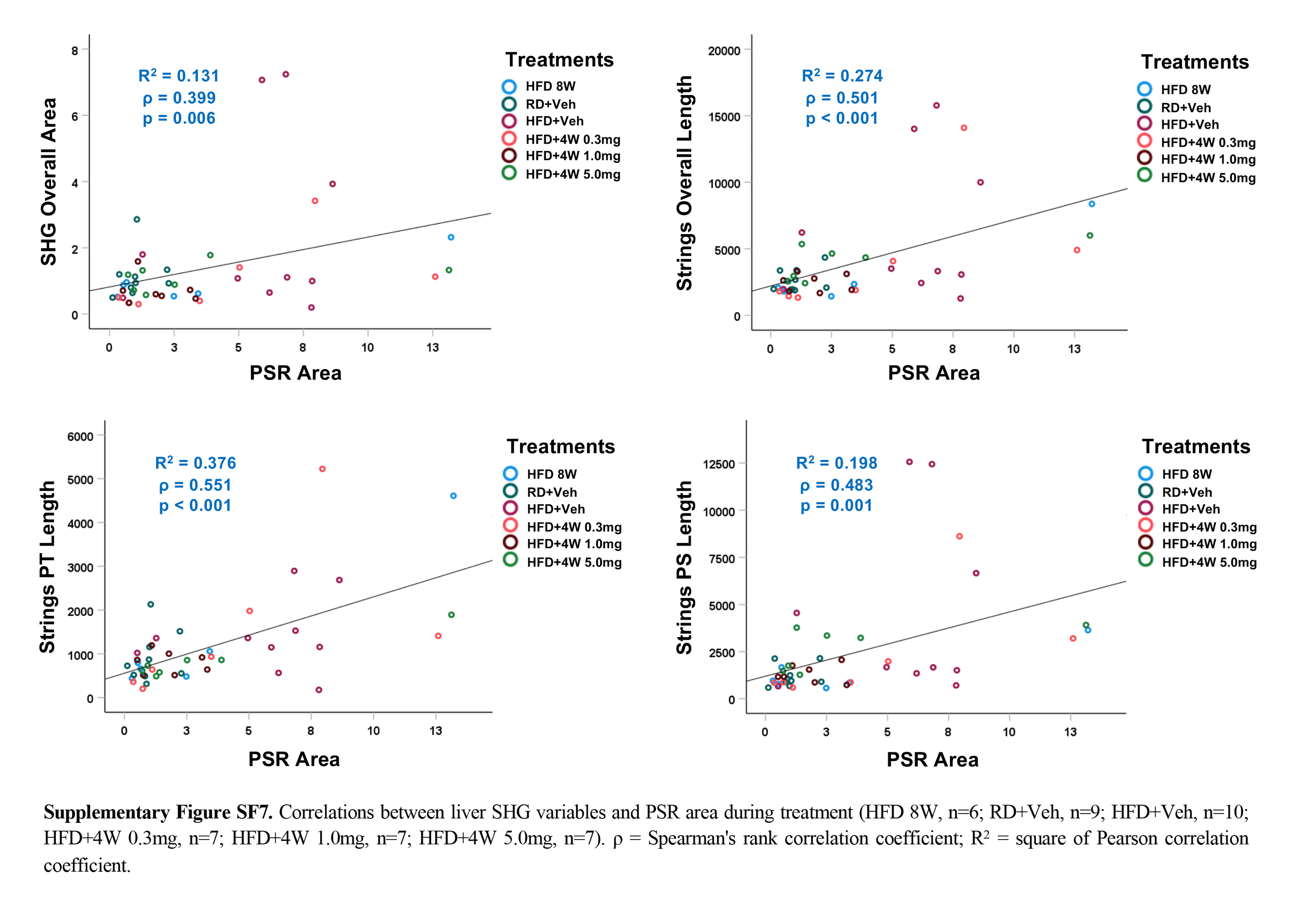

Supplement: Supplementary file 5 [file Image7.JPEG]

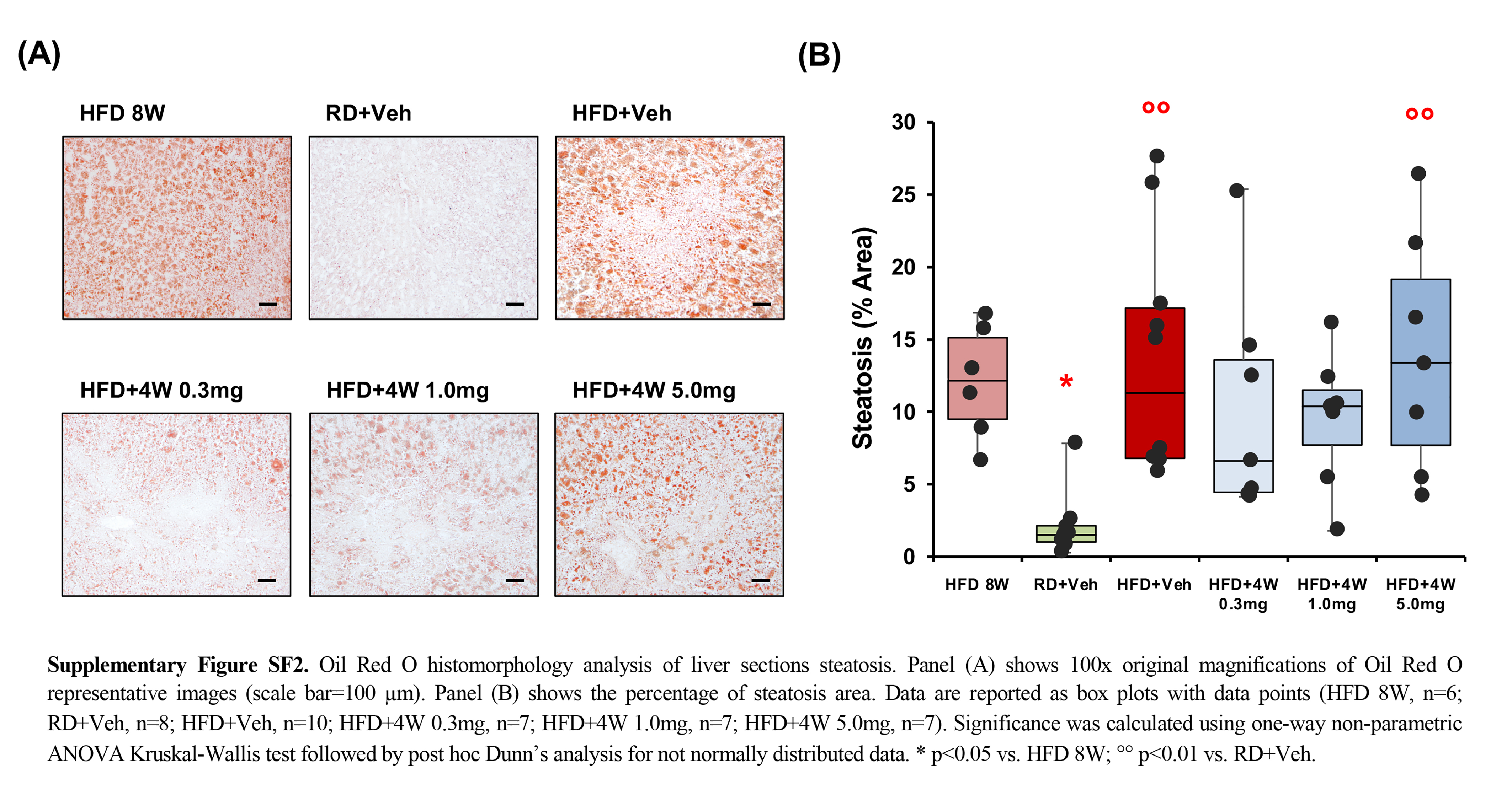

Supplement: Supplementary file 6 [file Image2.JPEG]

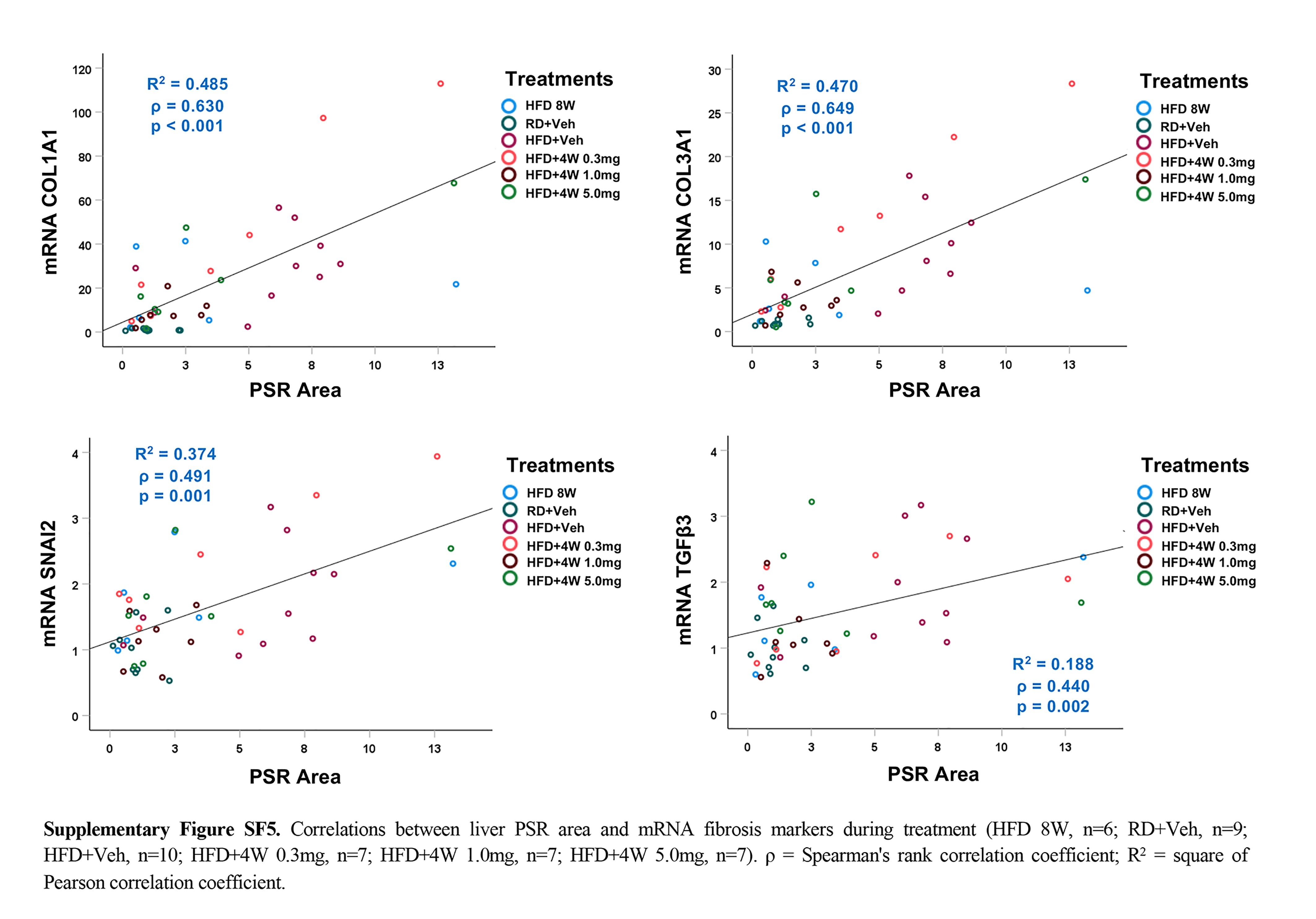

Supplement: Supplementary file 7 [file Image5.JPEG]

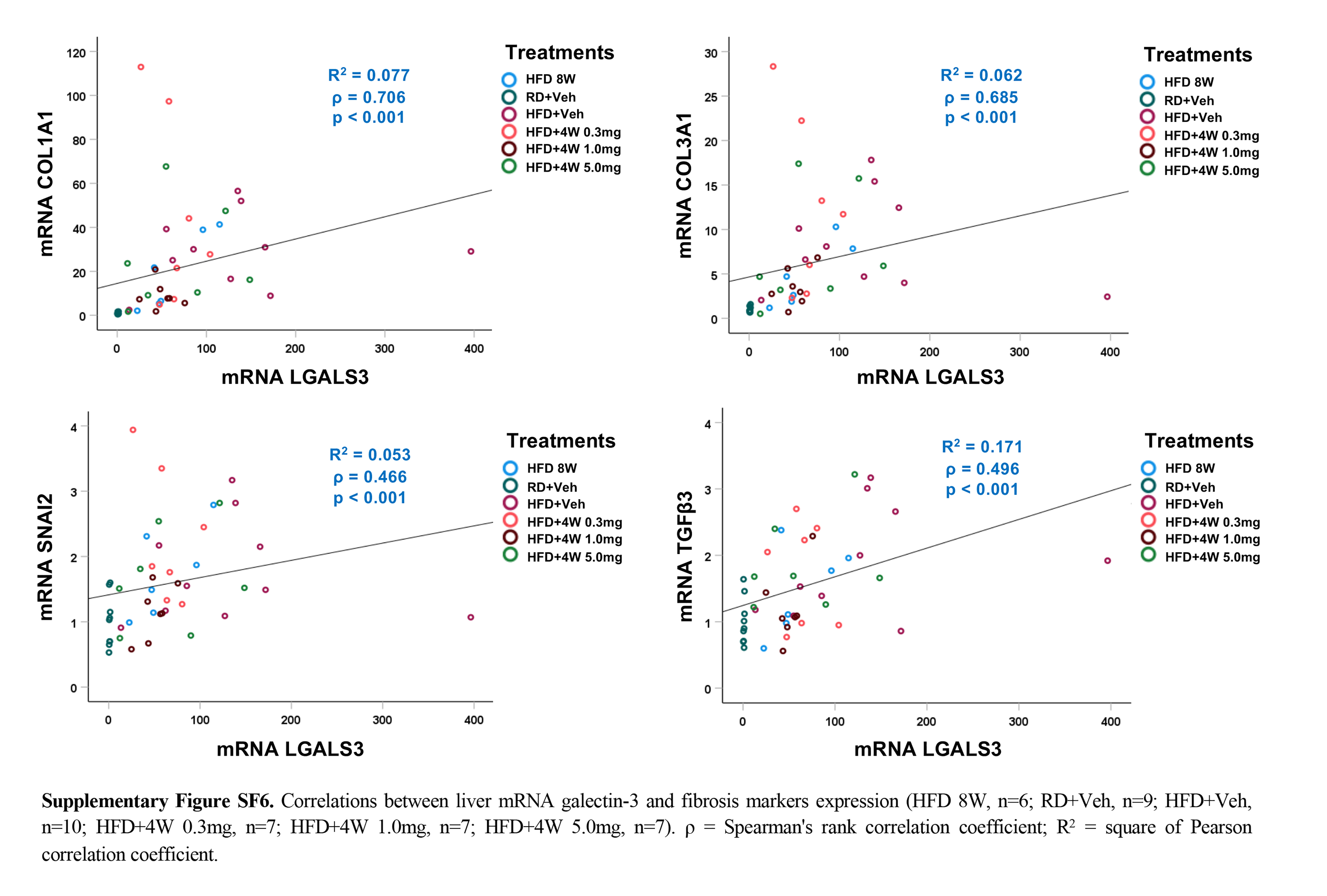

Supplement: Supplementary file 11 [file Image6.JPEG]
