# Supplementary material for: The galectin-3 inhibitor selvigaltin reduces liver inflammation and fibrosis in a high fat diet rabbit model of metabolic-associated steatohepatitis
Source: Front Pharmacol. 2024 Jul 31;15:1430109. doi: 10.3389/fphar.2024.1430109 (PMC11322497; doi:10.3389/fphar.2024.1430109)
Supplement: Supplementary file 2 [file Table1.docx]

**Supplementary Table ST1.** Clinical and biochemical data at sacrifice.

| **Variable** | **HFD 8W**  **(n=6)** | **RD+Veh**  **(n=9)** | **HFD+Veh**  **(n=10)** | **Sign.** | **HFD+4W**  **0.3mg**  **(n=7)** | **Sign.** | **HFD+4W**  **1.0mg**  **(n=7)** | **Sign.** | **HFD+4W 5.0mg**  **(n=7)** | **Sign.** |
| --- | --- | --- | --- | --- | --- | --- | --- | --- | --- | --- |
| *Glycaemia (gr/L)* | *1.60 ± 0.63* | *1.25 ± 0.30* | *1.63 ± 0.27* | *°* | *1.21 ± 0.15* | *^^* | *1.90 ± 0.87* |  | *1.43 ± 0.35* |  |
| **OGTT (iAUC)** | **197.05 ± 37.94** | **145.42 ± 18.41** | **188.98 ± 22.38** | **°°°** | **165.51 ± 20.71** |  | **189.37 ± 22.39** | **°°** | **165.00 ± 2.65** |  |
| **Plasma Cholesterol (mg/dL)** | **1,395.17 ± 389.45** | **27.78 ± 13.03** | **2,803.20 ± 689.56** | **°°°** | **2,277.43 ± 1,078.47** | **°°°** | **2,182.86 ± 776.09** | **°°°** | **1,546.43 ± 350.47** | **°°° ^^^** |
| *Plasma Triglycerides (mg/dL)* | *62.17 ± 7.68* | *74.33 ± 20.21* | *171.70 ± 94.34* | *°°°* | *374.00 ± 619.70* | *°°* | *157.29 ± 68.66* | *°°* | *191.43 ± 234.40* |  |
| **MAP (mmHg)** | **119.17 ± 11.58** | **94.02 ± 18.26** | **136.63 ± 23.65** | **°°°** | **144.91 ± 16.18** | **°°°** | **151.23 ± 26.21** | **°°°** | **141.04 ± 30.52** | **°°°** |
| **VAT Weight**  **(% of BW)** | **1.01 ± 0.25** | **0.91 ± 0.08** | **1.17 ± 0.18** | **°** | **0.67 ± 0.21** | **° ^^^** | **0.68 ± 0.24** | **° ^^^** | **0.87 ± 0.32** | **^^** |
| *Liver Weight*  *(% of BW)* | *3.83 ± 0.55* | *2.48 ± 0.38* | *3.89 ± 0.39* | *°°°* | *3.56 ± 0.28* | *°°* | *3.73 ± 0.32* | *°°°* | *3.56 ± 0.16* | *°* |
| **Tissue Triglycerides**  **(nmol/mg Liver)** | **11.50 ± 2.16** | **7.36 ± 2.64** | **15.15 ± 3.68** | **°°°** | **14.76 ± 2.26** | **°°°** | **14.34 ± 1.35** | **°°°** | **14.51 ± 1.49** | **°°°** |
| **Albumin (g/L) (#)** | **41.83 ± 1.17** | **41.50 ± 2.95** | **41.86 ± 3.08** |  | **38.29 ± 4.42** |  | **38.71 ± 2.63** |  | **39.29 ± 5.94** |  |
| **ALP (U/L) (#)** | **55.20 ± 26.55** | **59.33 ± 24.88** | **69.14 ± 29.45** |  | **69.43 ± 38.22** |  | **72.86 ± 65.69** |  | **49.57 ± 30.84** |  |
| **γGT (U/L)** | **13.00 ± 5.90** | **6.13 ± 2.02** | **35.00 ± 27.09** | **°** | **30.08 ± 22.90** | **°** | **27.71 ± 14.16** |  | **21.29 ± 13.11** | **°** |
| **AST (U/L)** | **59.83 ± 32.57** | **30.11 ± 8.82** | **103.30 ± 69.87** | **°°°** | **74.29 ± 51.50** | **°** | **59.00 ± 37.48** | **^** | **62.43 ± 15.80** |  |
| **ALT (U/L)** | **59.50 ± 57.26** | **30.11 ± 6.33** | **94.80 ± 55.18** | **°°°** | **45.71 ± 22.51** | **^^** | **58.14 ± 27.72** | **^** | **56.43 ± 20.02** | **^** |
| *Bilirubin (µM)* | *3.70 ± 4.90* | *2.53 ± 1.03* | *37.23 ± 46.87* | *°°°* | *17.91 ± 13.00* | *°°* | *9.53 ± 3.73* | *^* | *15.45 ± 9.05* | *°°* |

Data are expressed as mean±SD. iAUC, incremental area under the curve of glucose blood level during oral glucose tolerance test (OGTT); MAP, mean arterial pressure; VAT, visceral adipose tissue; BW, body weight; ALP, alkaline phosphatase; γGT, gamma-glutamyltransferase; AST, aspartate aminotransferase; ALT, alanine aminotransferase. Significance (Sign.): one-way parametric ANOVA test followed by post hoc Fisher’s Least Significant Difference (LSD) test for normally distributed data (in bold) and one-way non-parametric ANOVA Kruskal-Wallis test followed by post hoc Dunn’s analysis for not normally distributed data (in italic). No further test was performed when ANOVA test resulted not significant (#). ° p<0.05, °° p<0.01, °°° p<0.001 vs. RD+Veh; ^ p<0.05, ^^ p<0.01, ^^^ p<0.001 vs. HFD+Veh.
