# Supplementary material for: The galectin-3 inhibitor selvigaltin reduces liver inflammation and fibrosis in a high fat diet rabbit model of metabolic-associated steatohepatitis
Source: Front Pharmacol. 2024 Jul 31;15:1430109. doi: 10.3389/fphar.2024.1430109 (PMC11322497; doi:10.3389/fphar.2024.1430109)
Supplement: Supplementary file 8 [file Table2.DOCX]

**Supplementary Table ST2.** Contingency tables for inflammation, ballooning, and fibrosis scoring.

| **Analyses** | **Scores** | **HFD 8W**  **(n=6)** | **RD+Veh**  **(n=9)** | **Sign.** | **HFD+Veh**  **(n=10)** | **Sign.** | **HFD+4W**  **0.3mg**  **(n=7)** | **Sign.** | **HFD+4W**  **1.0mg**  **(n=7)** | **Sign.** | **HFD+4W 5.0mg**  **(n=7)** | **Sign.** |
| --- | --- | --- | --- | --- | --- | --- | --- | --- | --- | --- | --- | --- |
| **Inflammation** | **0** | 0.0 % | 88.9 % | ****** | 0.0 % | **°°°** | 14.3 % | **°°** | 14.3 % | **°** | 0.0 % | **°°°** |
|  | **1** | 66.6 % | 11.1 % |  | 80.0 % |  | 42.8 % |  | 71.4 % |  | 100.0 % |  |
|  | **2** | 16.7 % | 0.0 % |  | 10.0 % |  | 42.8 % |  | 14.3 % |  | 0.0 % |  |
|  | **3** | 16.7 % | 0.0 % |  | 10.0 % |  | 0.0 % |  | 0.0 % |  | 0.0 % |  |
| **Ballooning** | **0** | 0.0 % | 100.0 % | ******* | 0.0 % | *** °°°** | 0.0 % | *** °°°** | 0.0 % | **°°°** | 0.0 % | **°°°** |
|  | **1** | 100.0 % | 0.0 % |  | 50.0 % |  | 42.9 % |  | 85.7 % |  | 57.1 % |  |
|  | **2** | 0.0 % | 0.0 % |  | 50.0 % |  | 57.1 % |  | 14.3 % |  | 42.9 % |  |
| **Fibrosis**  **(Ishak Score)** | **0** | 16.7 % | 33.3 % |  | 0.0 % |  | 0.0 % | **°** | 0.0 % | **° ^ ç** | 0.0 % |  |
|  | **1** | 33.3 % | 33.3 % |  | 20.0 % |  | 14.3 % |  | 0.0 % |  | 0.0 % |  |
|  | **2** | 33.3 % | 22.2 % |  | 10.0 % |  | 0.0 % |  | 85.7 % |  | 42.8 % |  |
|  | **3** | 0.0 % | 11.1 % |  | 20.0 % |  | 57.1 % |  | 14.3 % |  | 42.8 % |  |
|  | **4** | 16.7 % | 0.0 % |  | 40.0 % |  | 28.6 % |  | 0.0 % |  | 14.3 % |  |
|  | **5** | 0.0 % | 0.0 % |  | 10.0 % |  | 0.0 % |  | 0.0 % |  | 0.0 % |  |

Significance (*Sign.*): Pearson’s Chi Square test. * p<0.05, ** p<0.01,*** p<0.001 vs. HFD 8W; ° p<0.05, °° p<0.01, °°° p<0.001 vs. RD+Veh; ^ p<0.05 vs. HFD+Veh; ç p<0.05 vs. HFD+4W 0.3mg.
