# Supplementary material for: The galectin-3 inhibitor selvigaltin reduces liver inflammation and fibrosis in a high fat diet rabbit model of metabolic-associated steatohepatitis
Source: Front Pharmacol. 2024 Jul 31;15:1430109. doi: 10.3389/fphar.2024.1430109 (PMC11322497; doi:10.3389/fphar.2024.1430109)
Supplement: Supplementary file 9 [file Table3.docx]

**Supplementary Table ST3.** Fibrosis and inflammation markers mRNA expression.

| **Fibrosis**  **Markers** | **HFD 8W**  **(n=6)** | **RD+Veh**  **(n=9)** | **HFD+Veh**  **(n=10)** | **Sign.** | **HFD+4W**  **0.3mg**  **(n=7)** | **Sign.** | **HFD+4W**  **1.0mg**  **(n=7)** | **Sign.** | **HFD+4W 5.0mg**  **(n=7)** | **Sign.** |
| --- | --- | --- | --- | --- | --- | --- | --- | --- | --- | --- |
| *COL1A1* | *19.34 ± 17.49* | *1.00 ± 0.44* | *29.10 ± 17.25* | *°°°* | *45.14 ± 43.26* | *°°°* | *9.00 ± 6.03* | *°* | *25.20 ± 23.86* | *°°°* |
| *COL3A1* | *4.76 ± 3.63* | *1.00 ± 0.32* | *8.37 ± 5.47* | *°°°* | *12.38 ± 9.89* | *°°°* | *3.49 ± 2.11* | *°* | *7.26 ± 6.59* | *°°* |
| **EDN1** | **1.24 ± 0.26** | **1.00 ± 0.17** | **1.45 ± 0.41** | **°°** | **1.08 ± 0.33** | **^** | **0.99 ± 0.28** | **^** | **1.09 ± 0.51** | **^** |
| *EDNRA* | *2.64 ± 1.41* | *1.00 ± 0.30* | *5.00 ± 4.63* | *°°°* | *3.66 ± 2.05* | *°°°* | *2.13 ± 1.20* | *°* | *3.04 ± 2.38* | *°°* |
| *EDNRB* | *1.94 ± 0.88* | *1.00 ± 0.31* | *2.01 ± 0.56* | *°°°* | *1.60 ± 0.42* | *°* | *1.58 ± 0.31* | *°* | *2.49 ± 2.19* | *°°°* |
| *FN1* | *1.21 ± 0.47* | *1.00 ± 0.18* | *1.34 ± 0.31* | *°* | *1.79 ± 0.76* | *°°* | *1.06 ± 0.22* |  | *1.51 ± 0.74* | *°* |
| *LGALS3* | *61.84 ± 35.53* | *1.00 ± 0.48* | *135.17 ± 105.23* | *°°°* | *63.90 ± 24.46* | *°°°* | *50.04 ± 15.84* | *°° ^* | *67.63 ± 53.87* | *°°* |
| **PAI-1** | **9.96 ± 7.51** | **1.00 ± 0.38** | **11.60 ± 11.90** | **°** | **4.91 ± 1.83** | **°** | **4.93 ± 2.25** | **°** | **5.06 ± 3.07** | **°** |
| *αSMA* | *3.02 ± 2.59* | *1.00 ± 0.64* | *2.43 ± 0.93* | *°* | *4.64 ± 4.05* | *°°* | *1.75 ± 1.34* |  | *3.17 ± 2.57* | *°* |
| *SNAI1 (#)* | *1.40 ± 1.98* | *1.00 ± 0.75* | *1.77 ± 1.32* |  | *2.05 ± 2.05* |  | *1.30 ± 1.21* |  | *2.32 ± 1.38* |  |
| *SNAI2* | *1.76 ± 0.70* | *1.00 ± 0.39* | *1.76 ± 0.78* | *°* | *2.28 ± 1.03* | *°°* | *1.15 ± 0.42* |  | *1.68 ± 0.79* | *°* |
| *TGFβ1* | *2.70 ± 0.92* | *1.00 ± 0.31* | *3.86 ± 1.58* | *°°°* | *4.06 ± 1.62* | *°°°* | *2.87 ± 1.14* | *°°* | *3.96 ± 2.80* | *°°°* |
| **TGFβ3** | **1.47 ± 0.68** | **1.00 ± 0.35** | **1.88 ± 0.82** | **°°** | **1.73 ± 0.80** | **°** | **1.20 ± 0.54** | **^** | **1.87 ± 0.71** | **°** |
| **Inflammation Markers** | **HFD 8W**  **(n=6)** | **RD+Veh**  **(n=9)** | **HFD+Veh**  **(n=10)** | **Sign.** | **HFD+4W**  **0.3mg**  **(n=7)** | **Sign.** | **HFD+4W**  **1.0mg**  **(n=7)** | **Sign.** | **HFD+4W 5.0mg**  **(n=7)** | **Sign.** |
| **CD4** | **1.48 ± 0.42** | **1.00 ± 0.42** | **2.36 ± 1.21** | **°°** | **1.96 ± 0.51** |  | **1.33 ± 0.66** | **^** | **2.60 ± 1.97** | **°°** |
| *COX-2* | *33.94 ± 39.42* | *1.00 ± 0.46* | *8.21 ± 6.36* | *°°°* | *8.60 ± 4.94* | *°°°* | *6.14 ± 3.84* | *°°* | *13.87 ± 22.12* | *°°°* |
| **FOXP3** | **1.46 ± 0.34** | **1.00 ± 0.32** | **4.46 ± 4.42** | **°°** | **2.85 ± 1.40** |  | **1.39 ± 0.43** | **^^** | **3.57 ± 1.87** | **°** |
| **IL1β (#)** | **0.94 ± 0.28** | **1.00 ± 0.98** | **1.60 ± 1.10** |  | **1.33 ± 0.37** |  | **1.18 ± 0.45** |  | **1.32 ± 0.62** |  |
| **IL2** | **2.57 ± 1.62** | **1.00 ± 0.51** | **2.37 ± 1.31** | **°** | **2.98 ± 1.16** | **°** | **1.85 ± 1.16** |  | **3.33 ± 2.66** | **°°** |
| *IL6* | *3.24 ± 2.22* | *1.00 ± 0.60* | *1.83 ± 1.39* |  | *2.43 ± 1.89* |  | *0.58 ± 0.22* | *^* | *1.59 ± 1.33* |  |
| *MCP1* | *18.94 ± 13.96* | *1.00 ± 0.77* | *6.95 ± 3.81* | *°°°* | *7.70 ± 6.93* | *°°* | *4.23 ± 1.50* | *°* | *5.47 ± 2.52* | *°°* |
| *RAGE* | *1.61 ± 0.47* | *1.00 ± 0.42* | *1.82 ± 0.89* | *°°* | *1.99 ± 1.10* | *°* | *1.14 ± 0.36* |  | *1.73 ± 0.67* | *°* |
| **TLR4** | **5.10 ± 1.79** | **1.00 ± 0.48** | **4.49 ± 2.44** | **°°°** | **3.89 ± 1.45** | **°°°** | **3.63 ± 0.90** | **°°** | **3.90 ± 1.30** | **°°°** |
| *TNFα* | *12.04 ± 1.43* | *1.00 ± 0.44* | *4.35 ± 1.85* | *°°°* | *4.39 ± 0.96* | *°°°* | *3.67 ± 2.21* | *°* | *3.80 ± 1.84* | *°°* |

Results are expressed as fold-change vs. RD+Veh and are reported as mean±SD. Significance (Sign.): one-way parametric ANOVA test followed by post hoc Fisher’s Least Significant Difference (LSD) test for normally distributed data (in bold) and one-way non-parametric ANOVA Kruskal-Wallis test followed by post hoc Dunn’s analysis for not normally distributed data (in italic). No further test was performed when ANOVA test resulted not significant (#). ° p<0.05, °° p<0.01, °°° p<0.001 vs. RD+Veh; ^ p<0.05, ^^ p<0.01 vs. HFD+Veh.
