# Supplementary material for: The galectin-3 inhibitor selvigaltin reduces liver inflammation and fibrosis in a high fat diet rabbit model of metabolic-associated steatohepatitis
Source: Front Pharmacol. 2024 Jul 31;15:1430109. doi: 10.3389/fphar.2024.1430109 (PMC11322497; doi:10.3389/fphar.2024.1430109)
Supplement: Supplementary file 10 [file Table4.docx]

**Supplementary Table ST4.** Fibrosis and inflammation markers protein expression.

| **Markers** | **HFD 8W**  **(n=4)** | **RD+Veh**  **(n=4)** | **HFD+Veh**  **(n=4)** | **Sign.** | **HFD+4W**  **0.3mg**  **(n=3)** | **Sign.** | **HFD+4W**  **1.0mg**  **(n=3)** | **Sign.** | **HFD+4W 5.0mg**  **(n=3)** | **Sign.** |
| --- | --- | --- | --- | --- | --- | --- | --- | --- | --- | --- |
| **Collagen**  **(µg/mg Liver)** | **40.36 ± 8.25** | **34.69 ± 1.58** | **63.70 ± 2.99** | **°°°** | **57.63 ± 2.73** | **°°°** | **45.25 ± 3.42** | **° ^^^** | **50.86 ± 5.22** | **°°° ^^** |
| *IL6*  *(pg/g Liver)* | *54.20 ± 14.56* | *13.75 ± 5.96* | *84.96 ± 7.59* | *°°°* | *76.12 ± 8.47* | *°* | *63.53 ± 20.39* |  | *64.27 ± 18.36* |  |
| **TNFα**  **(pg/mg Liver)** | **1.84 ± 0.19** | **0.61 ± 0.29** | **2.19 ± 0.63** | **°°°** | **1.61 ± 0.54** | **°** | **1.24 ± 0.58** | **^** | **1.33 ± 0.68** | **^** |

Results are expressed as mean±SD. Significance (Sign.): one-way parametric ANOVA test followed by post hoc Fisher’s Least Significant Difference (LSD) test for normally distributed data (in bold) and one-way non-parametric ANOVA Kruskal-Wallis test followed by post hoc Dunn’s analysis for not normally distributed data (in italic). ° p<0.05, °°° p<0.001 vs. RD+Veh; ^ p<0.05, ^^ p<0.01, ^^^ p<0.001 vs. HFD+Veh.
